# Supplementary material for: New Methyl Threonolactones and Pyroglutamates of Spilanthes acmella (L.) L. and Their Bone Formation Activities
Source: Molecules. 2020 May 28;25(11):2500. doi: 10.3390/molecules25112500 (PMC7321231; doi:10.3390/molecules25112500)

Supplementary Data

New methyl threonolactones and pyroglutamates of *Spilanthes acmella* Murr. and their bone formation activities

Retno Widyowati [1,2,*], Melanny Ika Sulistyowaty [2,3], Nguyen Hoang Uyen [2], Sachiko Sugimoto [2], Yoshi Yamano [2], Hideaki Otsuka [4], and Katsuyoshi Matsunami [2,*]

[1] Department of Pharmacognosy and Phytochemistry, Faculty of Pharmacy, Airlangga University, Gedung Nanizar Zaman Joenoes, Kampus C Unair, Surabaya, 60115, Indonesia; rr-retno-w@ff.unair.ac.id (R.W.)

[2] Graduate School of Biomedical & Health Sciences, Hiroshima University; 1-2-3 Kasumi, Minami-ku, Hiroshima, 734-8551, Japan; melanny-i-s@ff.unair.ac.id (M.I.S.); d173259@hiroshima-u.ac.jp (N.H.U.); ssugimot@hiroshima-u.ac.jp (S.S.); yamano@hiroshima-u.ac.jp (Y.Y.); matunami@hiroshima-u.ac.jp (K.M.)

[3] Department of Pharmaceutical Chemistry, Faculty of Pharmacy, Airlangga University, Gedung Nanizar Zaman Joenoes, Kampus C Unair, Surabaya, 60115, Indonesia; melanny-i-s@ff.unair.ac.id (M.I.S.)

[4] Graduate School of Pharmacy, Yasuda Women's University, Hiroshima, Japan; otsuka-h@yasuda-u.ac.jp (H.O.)

* Correspondence: matunami@hiroshima-u.ac.jp; Tel.: +81-82-257-5335 (K.M.)
rr-retno-w@ff.unair.ac.id; Tel.: +62-816-1588-6978 (R.W.)

Contents:

Figure S1: 1H NMR of compound **1** in 600MHz, CD_3OD

Figure S2: 1H NMR (3.237 & 3.238 ppm) of compound **1** in 600MHz, CD_3OD

Figure S3: 1H NMR (3.34 & 3.36 ppm) of compound **1** in 600MHz, CD_3OD

Figure S4: 13C & DEPT135 NMR of compound **1** in 150 MHz, CD_3OD

Figure S5: COSY of compound **1** in 600 MHz, CD_3OD

Figure S6: HSQC of compound **1** in 150 & 600 MHz, CD_3OD

Figure S7: HMBC of compound **1** in 150 and 600 MHz, CD_3OD

Figure S8: PS-NOESY of compound **1** in 600 MHz, CD_3OD

Figure S9: 1H NMR of compound **2** in 600 MHz, CD_3OD

Figure S10: 13C NMR of compound **2** in 150 MHz, CD_3OD

Figure S11: COSY of compound **2** in 600 MHz, CD_3OD

Figure S12: HSQC of compound **2** in 150 and 600 MHz, CD_3OD

Figure S13: HMBC of compound **2** in 150 and 600 MHz, CD_3OD

Figure S14: 1H NMR of compound **3** in 600 MHz, CD_3OD

Figure S15: 1H NMR (2.31 & 2.37 ppm) of compound **3** in 600 MHz, CD_3OD

Figure S16: 1H NMR (3.86 ppm) of compound **3** in 600 MHz, CD_3OD

Figure S17: 1H NMR (4.27 & 4.29 ppm) of compound **3** in 600 MHz, CD_3OD

Figure S18: 13C NMR of compound **3** in 150 MHz, CD_3OD

Figure S19: COSY of compound **3** in 600 MHz, CD_3OD

Figure S20: HSQC of compound **3** in 150 and 600 MHz, CD_3OD

Figure S21: HMBC of compound **3** in 150 and 600 MHz, CD_3OD

Figure S22: 1H NMR of compound **4** in 600 MHz, CD_3OD

Figure S23: 1H NMR (1.62 & 1.67 ppm) of compound **4** in 600 MHz, CD_3OD

Figure S24: 1H NMR (2.17 ppm) of compound **4** in 600 MHz, CD_3OD

Figure S25: 1H NMR (2.31 & 2.37 ppm) of compound **4** in 600 MHz, CD_3OD

Figure S26: 1H NMR (2.49 ppm) of compound **4** in 600 MHz, CD_3OD

Figure S27: 1H NMR (3.65 & 3.68 ppm) of compound **4** in 600 MHz, CD_3OD

Figure S28: 1H NMR (3.89 ppm) of compound **4** in 600 MHz, CD_3OD

Figure S29: 13C NMR of compound **4** in 150 MHz, CD_3OD

Figure S30: COSY of compound **4** in 600 MHz, CD_3OD

Figure S31: HSQC of compound **4** in 150 and 600 MHz, CD_3OD

Figure S32: HMBC of compound **4** in 150 and 600 MHz, CD_3OD

Figure S1 1H NMR of compound 1 in 600MHz, CD_3OD

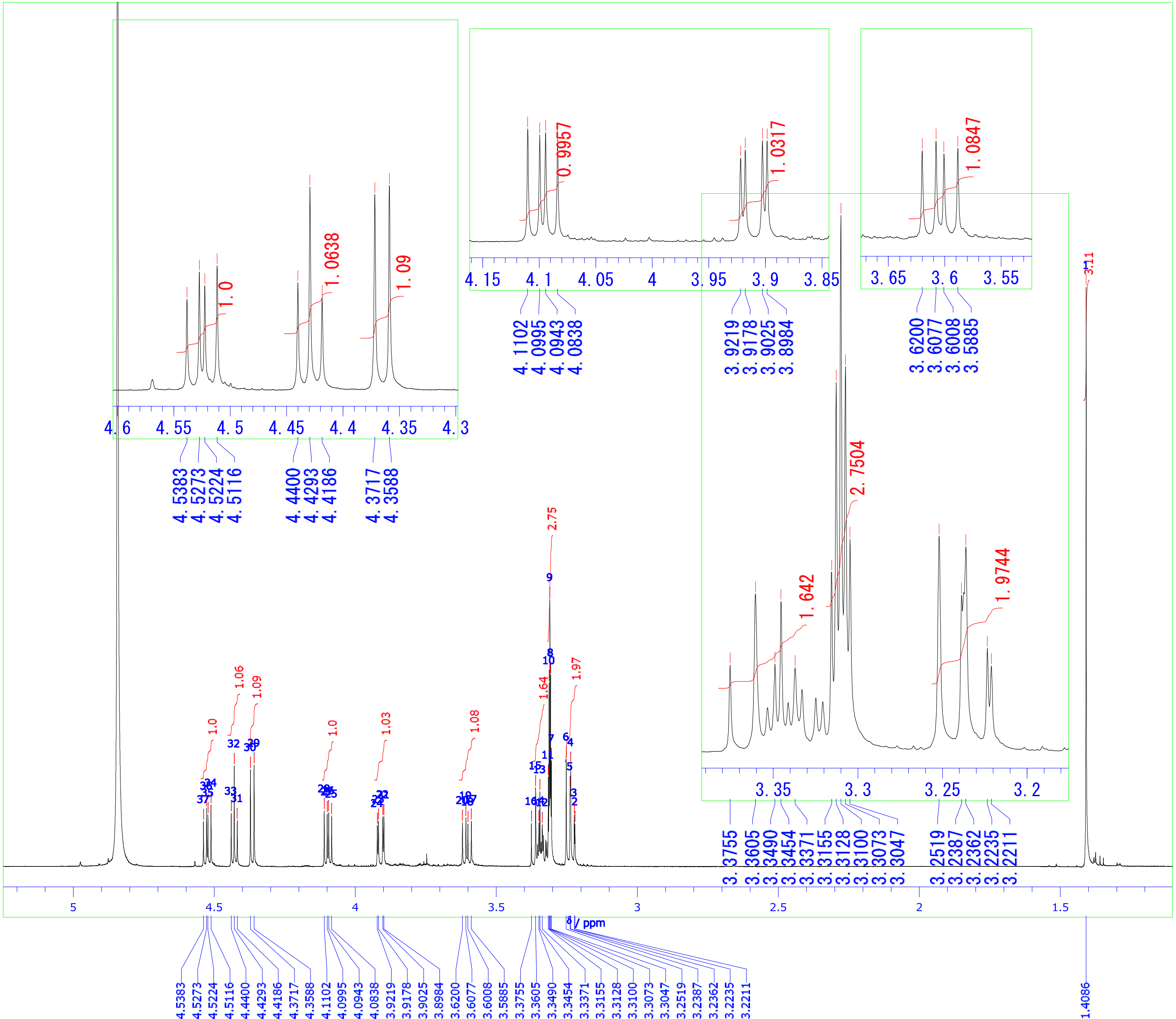

DFILE D:¥5 教室員研究¥08 RETNO WIDYOWATI ¥7RETNO 化合物NMR¥CHECK済み¥SAB 11-1-6 (CPD G)¥1R.RM1
DATIM 23/Nov/2014 12:04:11
COMNT
Sab 11-1-6 MeOD 10.1 mg H

OBNUC	^{1}H
EXMOD	ZG30
OBFRQ	600.13 MHz
OBSET	0.0 kHz
OBFIN	9987.786 Hz
POINT	65536
FREQU	12376.24 Hz
SCANS	8
ACQTM	5.2953 s
PD	1.0 s
PW1	10.0 μs
IRNUC	??
PROBHD	5 MM TXI 1H-13C/15N-D XYZ-GRD Z 8
PULSPRG	ZG30
GRDPROG	
CTEMP	26.85 ℃
SLVNT	MEOD
EXREF	3.31 ppm
BF	0.0944 Hz
WINDOW	Exponential

Figure S2 1H NMR (3.237 & 3.238 ppm) of compound 1 in 600MHz, CD_3OD

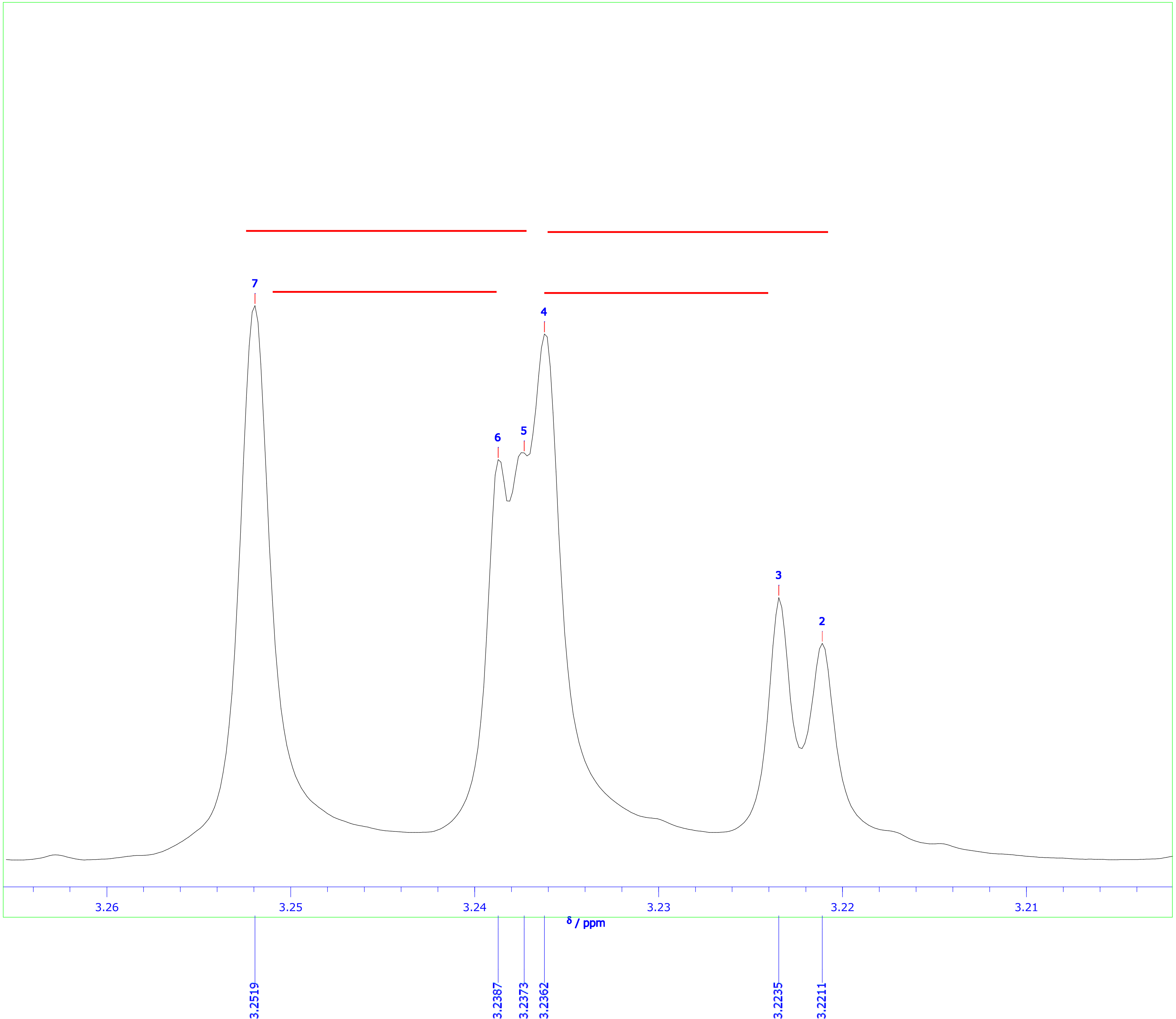

DFILE D:¥5 教室員研究¥08 RETNO WIDYOWATI ¥7RETNO 化合物NMR¥CHECK済み¥SAB 11-1-6 (CPD G)¥1R.RM1
DATIM 23/Nov/2014 12:04:11
COMNT
Sab 11-1-6 MeOD 10.1 mg H

OBNUC	^{1}H
EXMOD	ZG30
OBFRQ	600.13 MHz
OBSET	0.0 kHz
OBFIN	9987.786 Hz
POINT	65536
FREQU	12376.24 Hz
SCANS	8
ACQTM	5.2953 s
PD	1.0 s
PW1	10.0 μs
IRNUC	??
PROBHD	5 MM TXI 1H-13C/15N-D XYZ-GRD Z 8
PULSPRG	ZG30
GRDPROG	
CTEMP	26.85 ℃
SLVNT	MEOD
EXREF	3.31 ppm
BF	0.0944 Hz
WINDOW	Exponential

Figure S3 1H NMR (3.34 & 3.36 ppm) of compound 1 in 600MHz, CD_3OD

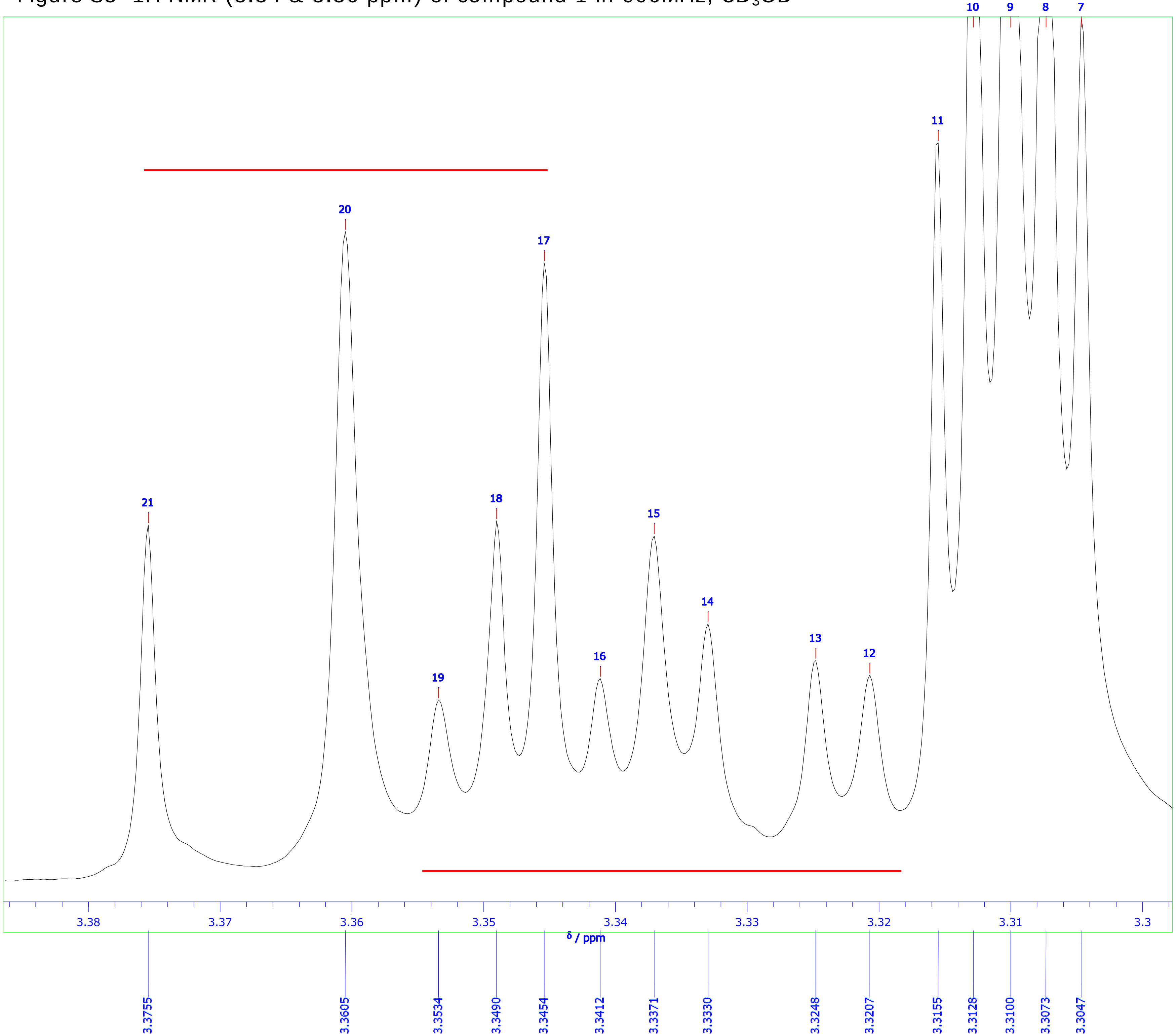

DFILE D:¥5 教室員研究¥08 RETNO WIDYOWATI ¥7RETNO 化合物NMR¥CHECK済み¥SAB 11-1-6 (CPD G)¥1R.RM1
DATIM 23/Nov/2014 12:04:11
COMNT Sab 11-1-6 MeOD 10.1 mg H

OBNUC	^1H
EXMOD	ZG30
OBFRQ	600.13 MHz
OBSET	0.0 kHz
OBFIN	9987.786 Hz
POINT	65536
FREQU	12376.24 Hz
SCANS	8
ACQTM	5.2953 s
PD	1.0 s
PW1	10.0 μs
IRNUC	??
PROBHD	5 MM TXI 1H-13C/15N-D XYZ-GRD Z 8
PULSPRG	ZG30
GRDPROG	
CTEMP	26.85 ℃
SLVNT	MEOD

Figure S4 13C & DEPT135 NMR of compound 1 in 150 MHz, CD_3OD

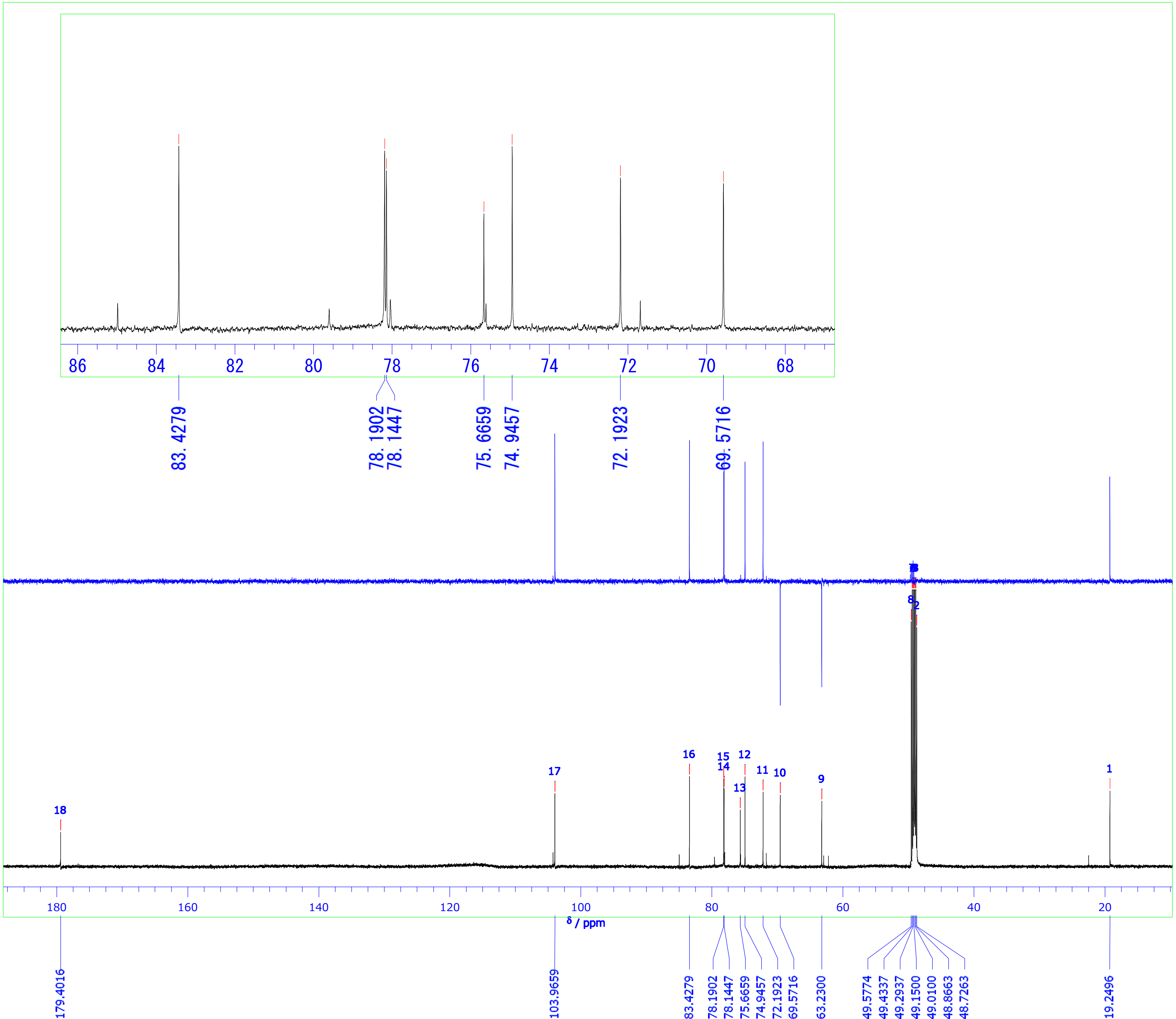

DFILE D:¥5 教室員研究¥08 RETNO WIDYOWATI ¥7RETNO 化合物NMR¥CHECK済み¥SAB 11-1-6 (CPD G)¥13C.RM1
DATIM 24/Nov/2014 05:25:23
COMNT
Sab 11-1-6 MeOD 10.1 mg c

OBNUC	^{13}C
EXMOD	ZGPG30
OBFRQ	150.9 MHz
OBSET	0.0 kHz
OBFIN	10000.86 Hz
POINT	32768
FREQU	35971.22 Hz
SCANS	10000
ACQTM	0.911 s
PD	2.0 s
PW1	15.0 μs
IRNUC	??
PROBHD	5 MM TXI 1H-13C/15N-D XYZ-GRD Z 8
PULSPRG	ZGPG30
GRDPROG	
CTEMP	26.85 ℃
SLVNT	MEOD
EXREF	49.15 ppm

Figure S5 COSY of compound 1 in 600 MHz, CD_3OD

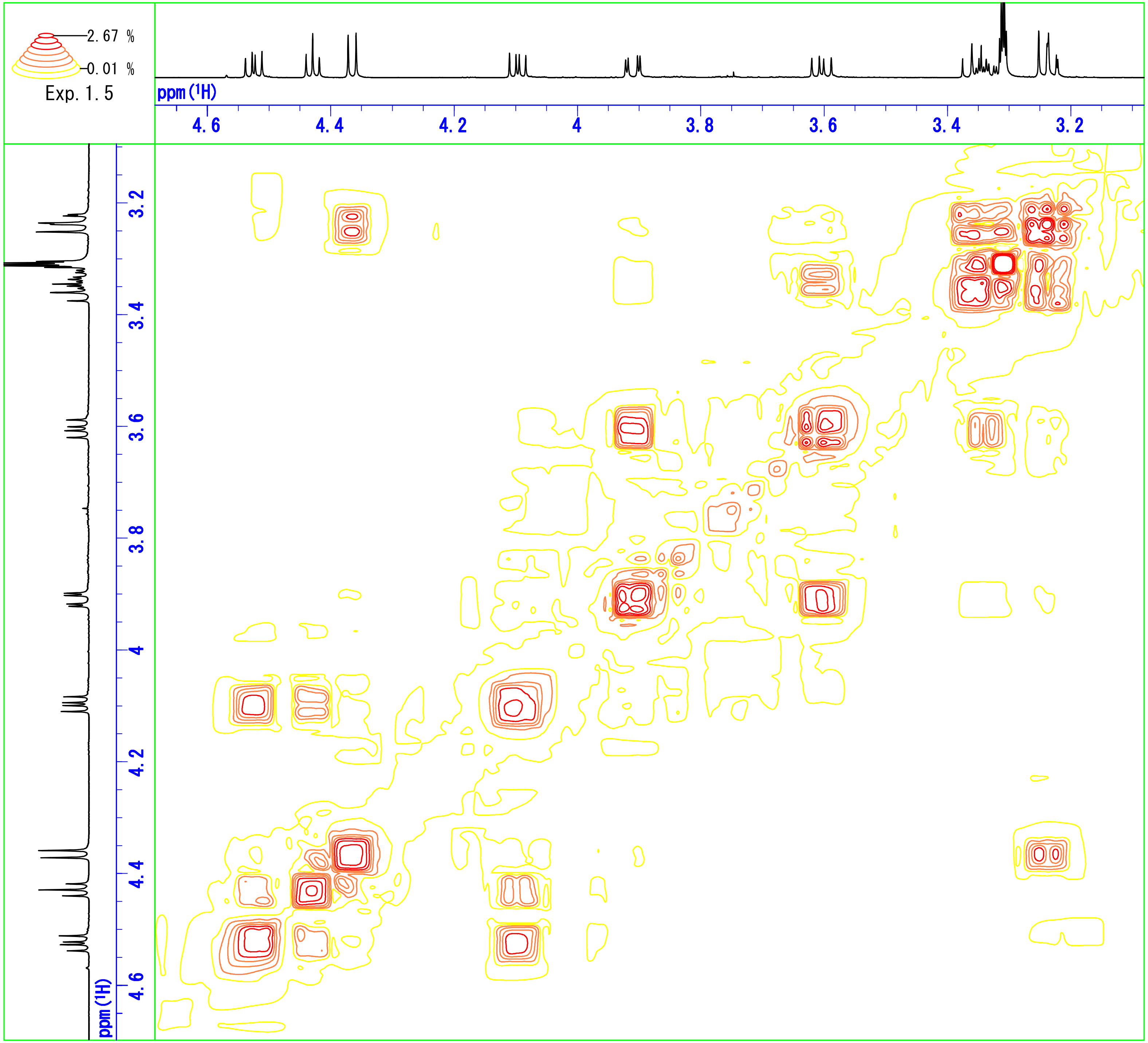

DFILE D:\5 教室員研究\08 RETNO WIDYOWATI\7RETNO 化合物NMR\CHECK済み\SAB 11-1-6 (CPD G)\COSY.RM2
ORGFILE C:\Users\PrimeVF17A\Desktop\RETNO NMR\Sab 11-1-6 (Cpd G)\42\pdata\1\2rr
DATIM 23/Nov/2014 14:36:56
COMNT
Sab 11-1-6 MeOD 10.1 mg cosy

EXMOD	V_HOMO(COSYGPQF)
OBNUC	^{1}H
OBFRQ	600.124 MHz
OBSET	0.0 kHz
OBFIN	10011.91 Hz
POINT	1024
FREQU	8012.82 Hz
CLPNT	1024
CLFRQ	8012.82 Hz
SCANS	15
ACQTM	0.1278 s
PD	1.4869 s
PW1	10.0 µs
PW2	10.0 µs
PW3	20.0 µs
PI1	1.4869 ms
PI2	0.0 ms
PI3	0.0 ms
IRNUC	^{1}H
CTEMP	26.85 °C
SLVNT	MEOD
EXREF	-0.6739 ppm
CLEXR	-0.6739 ppm
RGAIN	64
1DSPC(F1/F2)	G:\NOV23-2014RETNO\40\PDATA\1\1I
PRNT_DATE	2020/Apr/29 16:48:09
OPERT	

Figure S6 HSQC of compound 1 in 150 & 600 MHz, CD_3OD

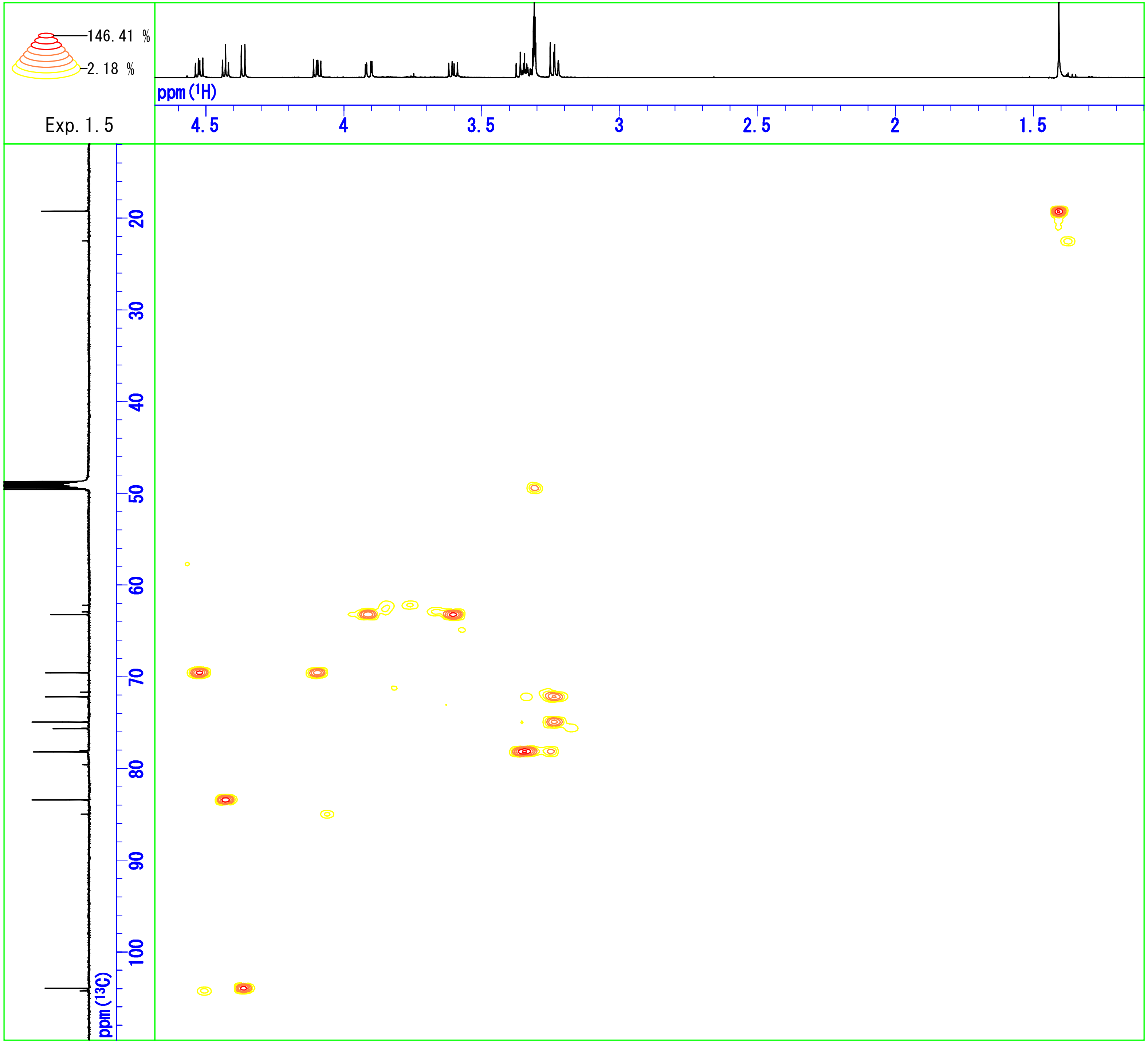

DFILE D:\5 教室員研究\08 RETNO WIDYOWATI\7RETNO 化合物NMR\CHECK済み\SAB 11-1-6 (CPD G)\HSQC.RM2
ORGFILE C:\Users\PrimeVF17A\Desktop\RETNO NMR\Sab 11-1-6 (Cpd G)\44\pdata\1\2rr
DATIM 23/Nov/2014 19:26:58
COMNT
Sab 11-1-6 MeOD 10.1 mg hsqc

EXMOD	VPH_HETERO(HSQCETGP)
OBNUC	^{1}H
OBFRQ	600.124 MHz
OBSET	0.0 kHz
OBFIN	10011.91 Hz
POINT	1024
FREQU	8012.82 Hz
CLPNT	1024
CLFRQ	25000.0 Hz
SCANS	15
ACQTM	0.1278 s
PD	1.5 s
PW1	10.0 μs
PW2	10.0 μs
PW3	20.0 μs
PI1	1.5 ms
PI2	0.0035 ms
PI3	0.0023 ms
IRNUC	^{13}C
CTEMP	26.85 °C
SLVNT	MEOD
EXREF	-0.6739 ppm
CLEXR	-6.5465 ppm
RGAIN	18390
1DSPC(F1)	G:\NOV23-2014RETNO\45\PDATA\1\1I
1DSPC(F2)	G:\NOV23-2014RETNO\40\PDATA\1\1I
PRNT_DATE	2020/Apr/29 16:50:50
OPERT	

Figure S7 HMBC of compound 1 in 150 and 600 MHz, CD_3OD

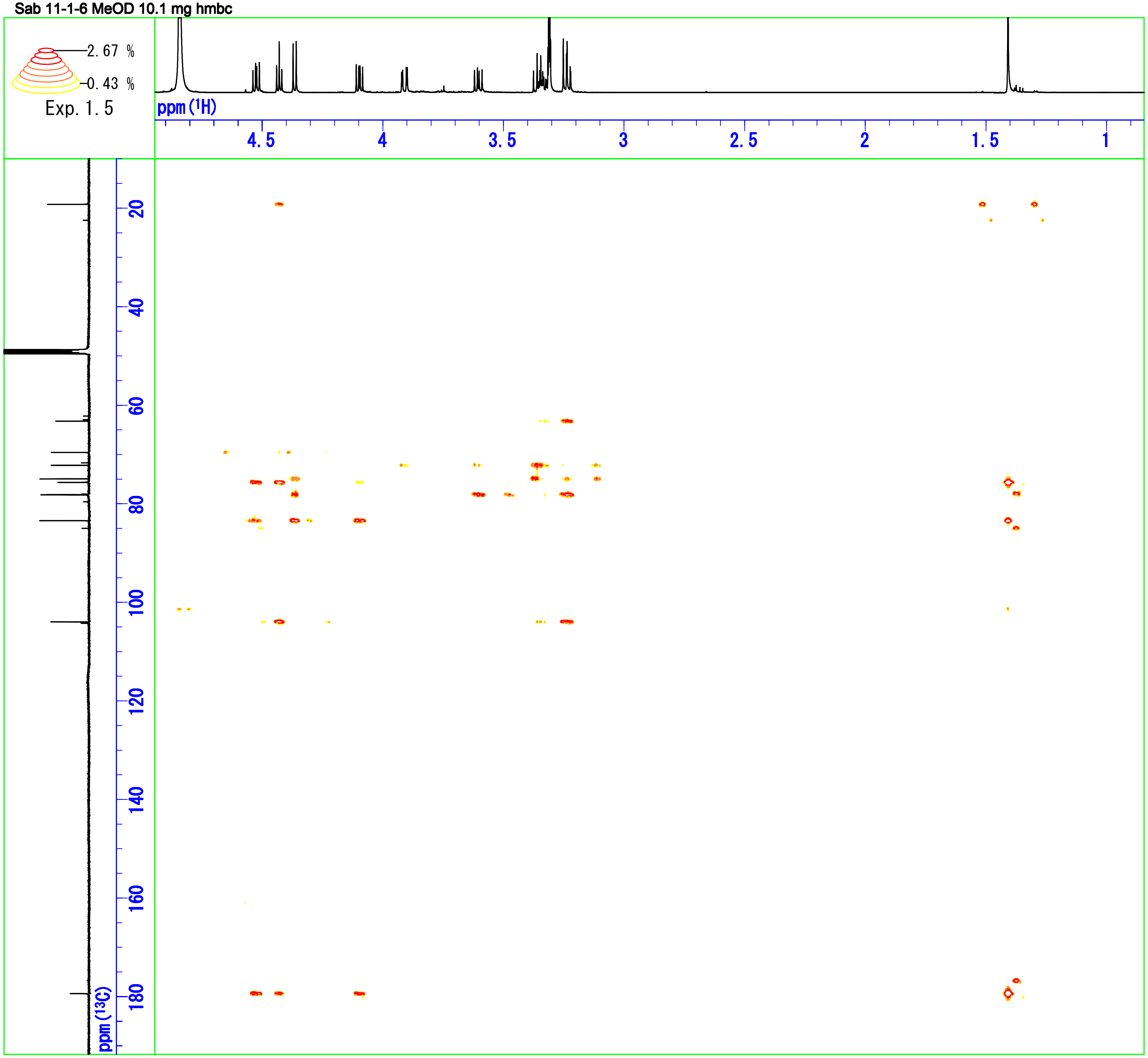

DFILE D:\5 教室員研究\08 RETNO WIDYOWATI\7RETNO 化合物NMR\CHECK済み\SAB 11-1-6 (CPD G)\HMBC.RM2
DATIM 23/Nov/2014 15:30:56
COMNT
Sab 11-1-6 MeOD 10.1 mg hmbc
EXMOD V_HETERO(HMBCGPLPNDQF)
-----F2 Parameter-------
F2NUC 1H
F2FRQ 600.133780819 MHz
F2PNT 2048
F2RES 3.802814 Hz
F2REF -0.20722 ppm
F2REP 0
-----F1 Parameter-------
F1NUC 13C
F1FRQ 600.133780819 MHz
F1PNT 1024
F1RES 32.77055 Hz
F1REF -9.5882 ppm
F1REP 0
---Aquisition Parameter------
SCANS 15
ACQTM 0.263 s
PD 1.5 s
PW1 10.0 μs
PW2 10.0 μs
PW3 20.0 μs
PI1 1.5 ms
PI2 0.0034 ms
PI3 0.0023 ms
CTEMP 26.85 °C
SLVNT MEOD
RGAIN 16384
1DSPC(F1) G:\Sab 11-1-6\13C.rm1
1DSPC(F2) G:\Sab 11-1-6\1R.rm1

Operator

Figure S8 PS-NOESY of compound 1 in 600 MHz, CD_3OD

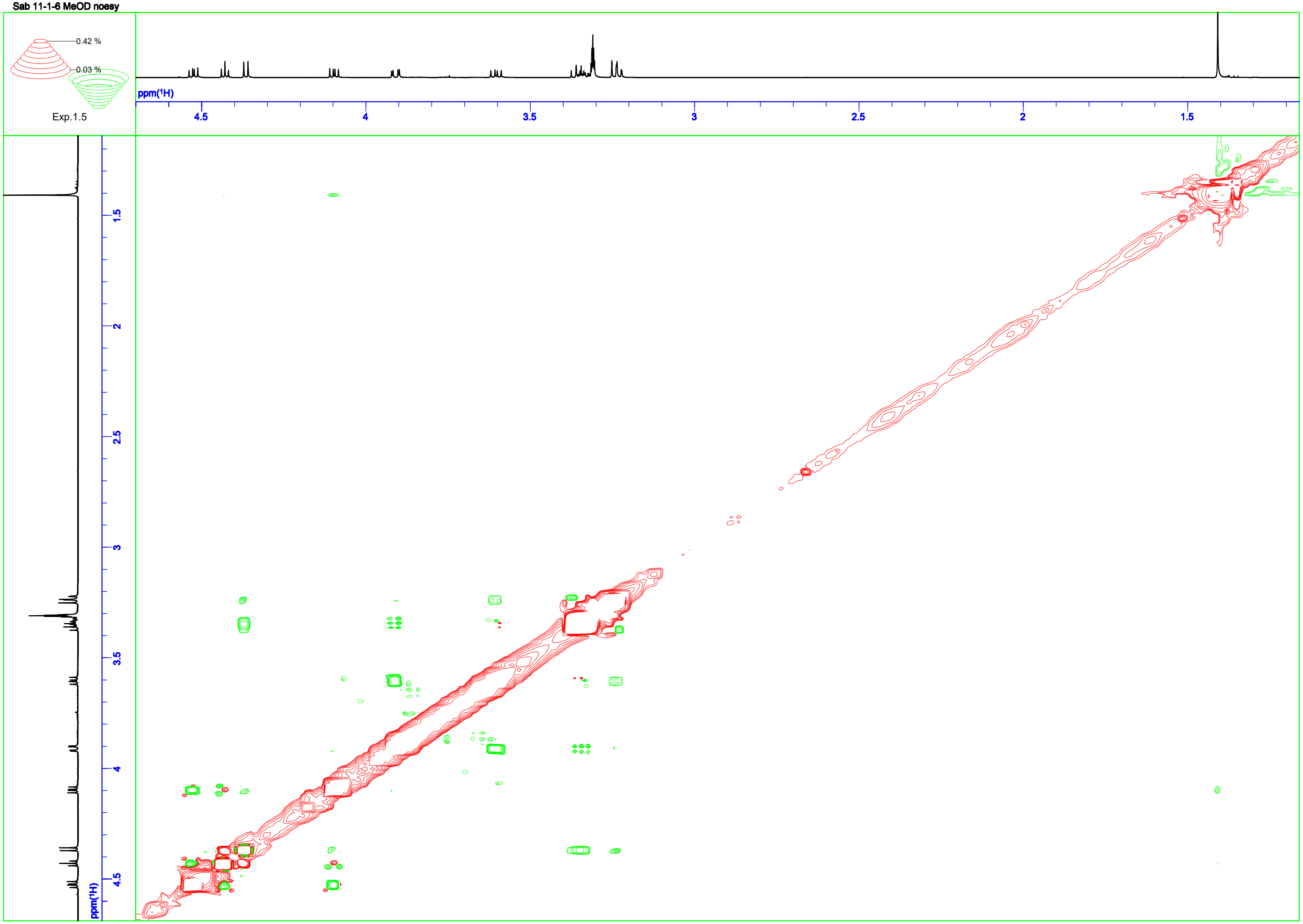

Figure S9 1H NMR of compound 2 in 600 MHz, CD_3OD

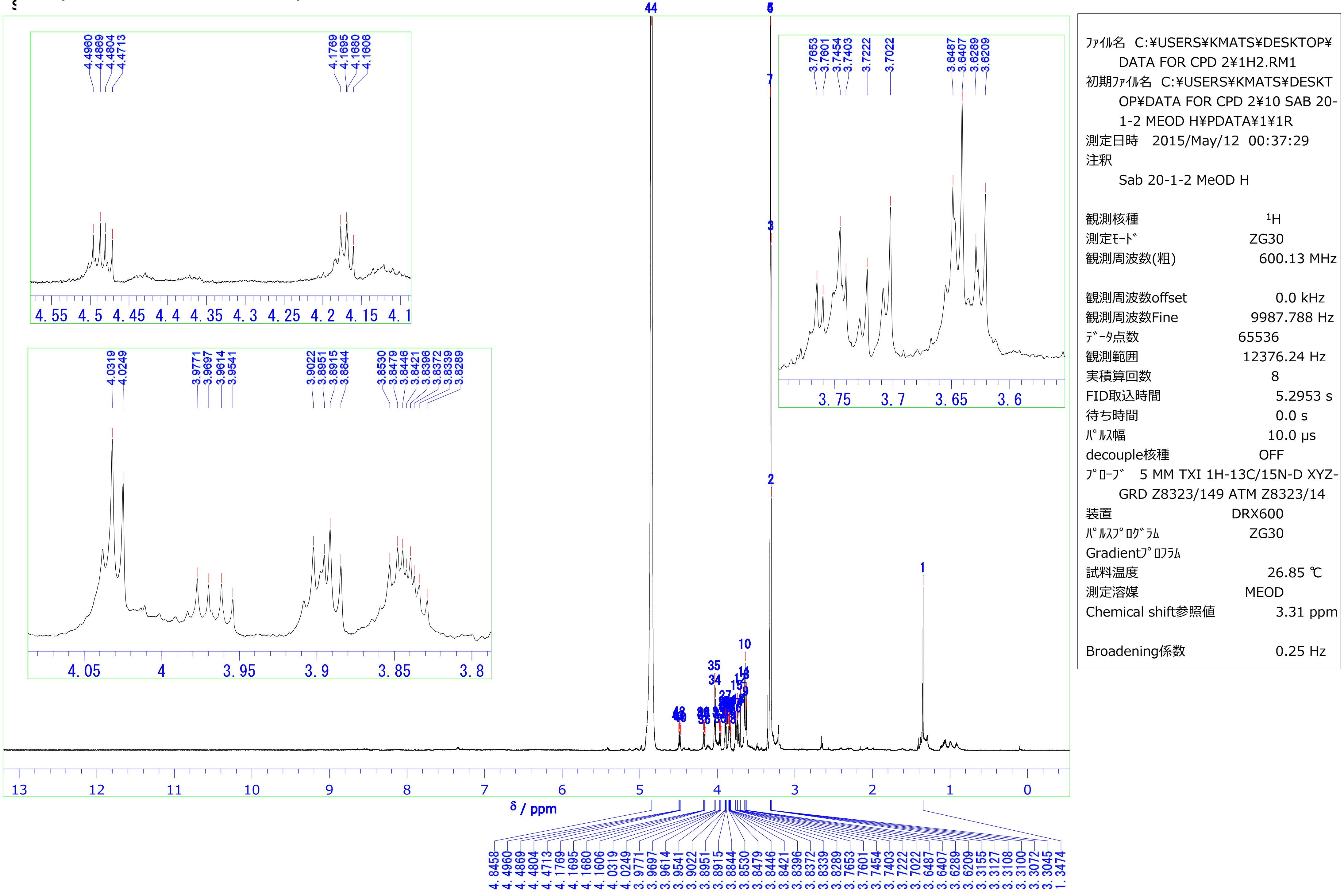

Figure S10 13C NMR of compound 2 in 150 MHz, CD_3OD

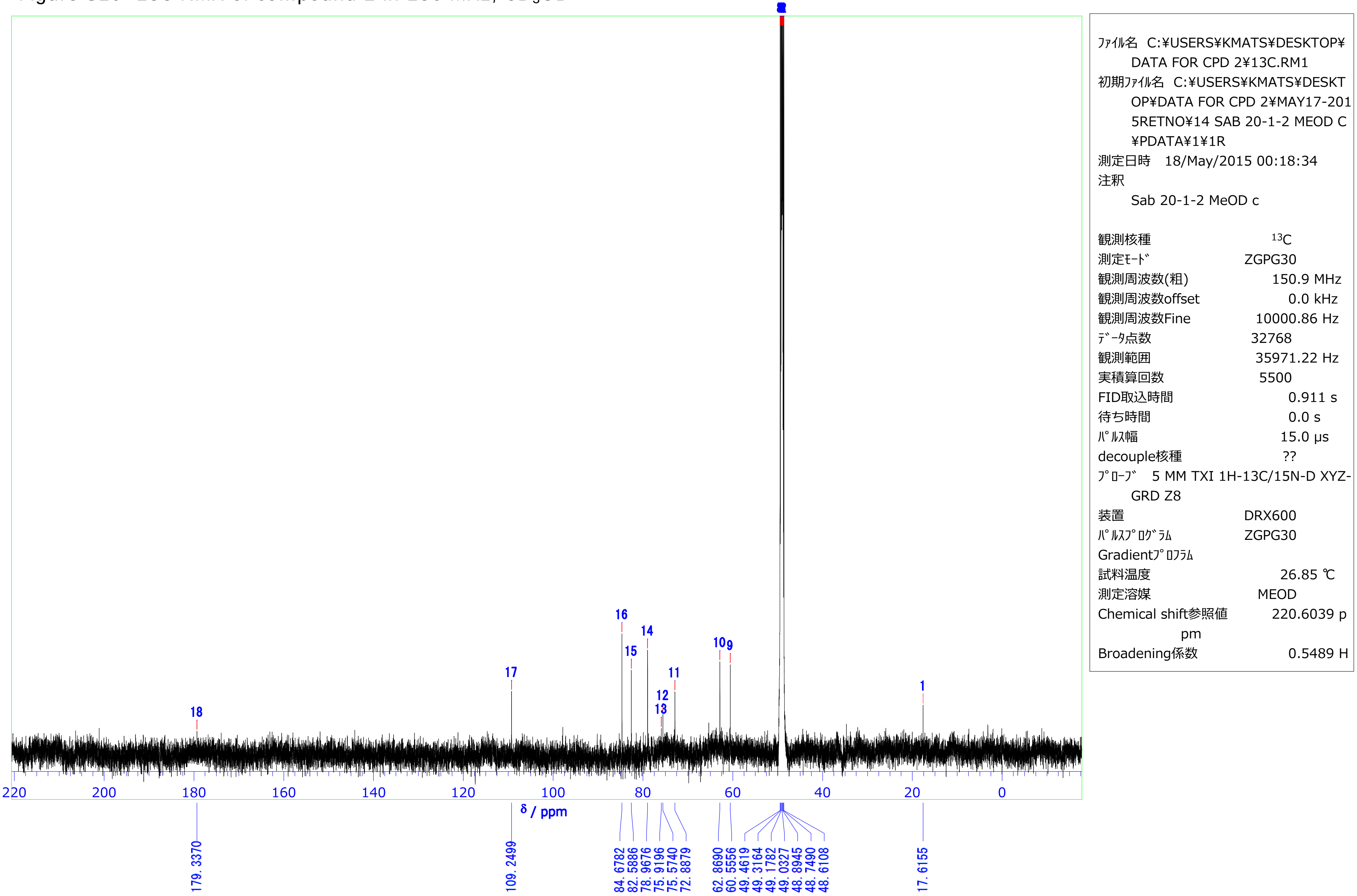

Figure S11 COSY of compound 2 in 600 MHz, CD_3OD

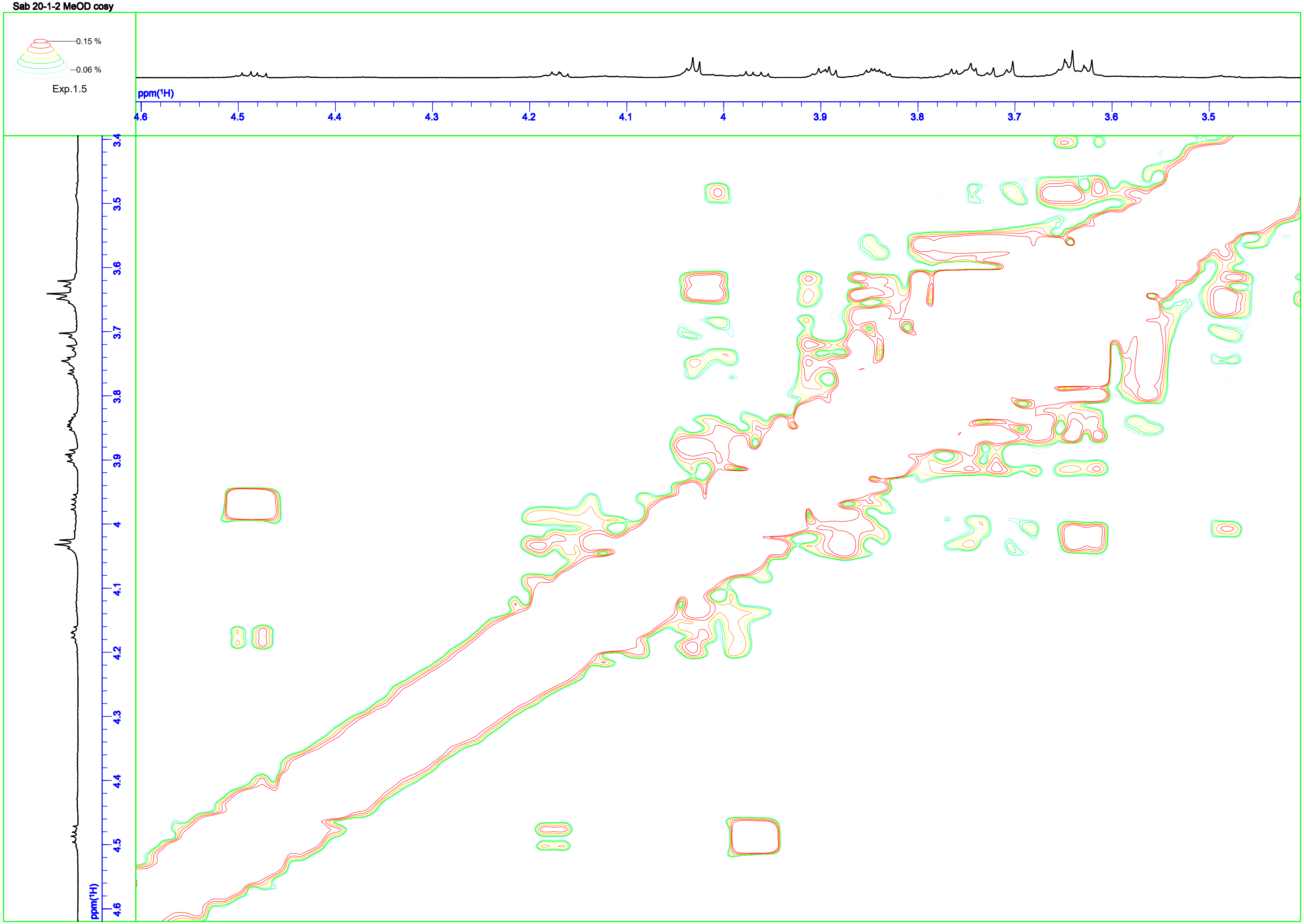

Figure S12 HSQC of compound 2 in 150 and 600 MHz, CD_3OD

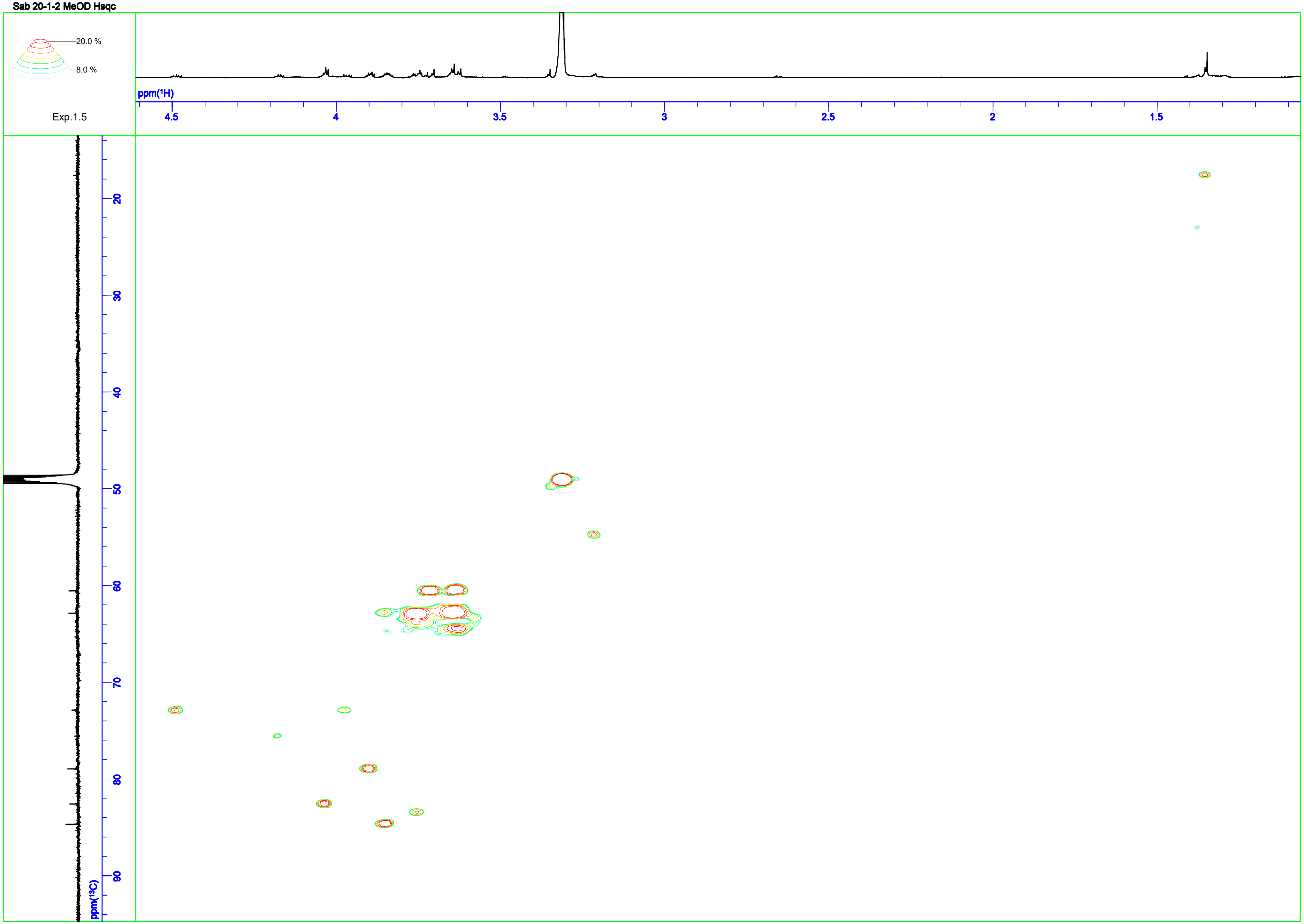

Figure S13 HMBC of compound 2 in 150 and 600 MHz, CD_3OD

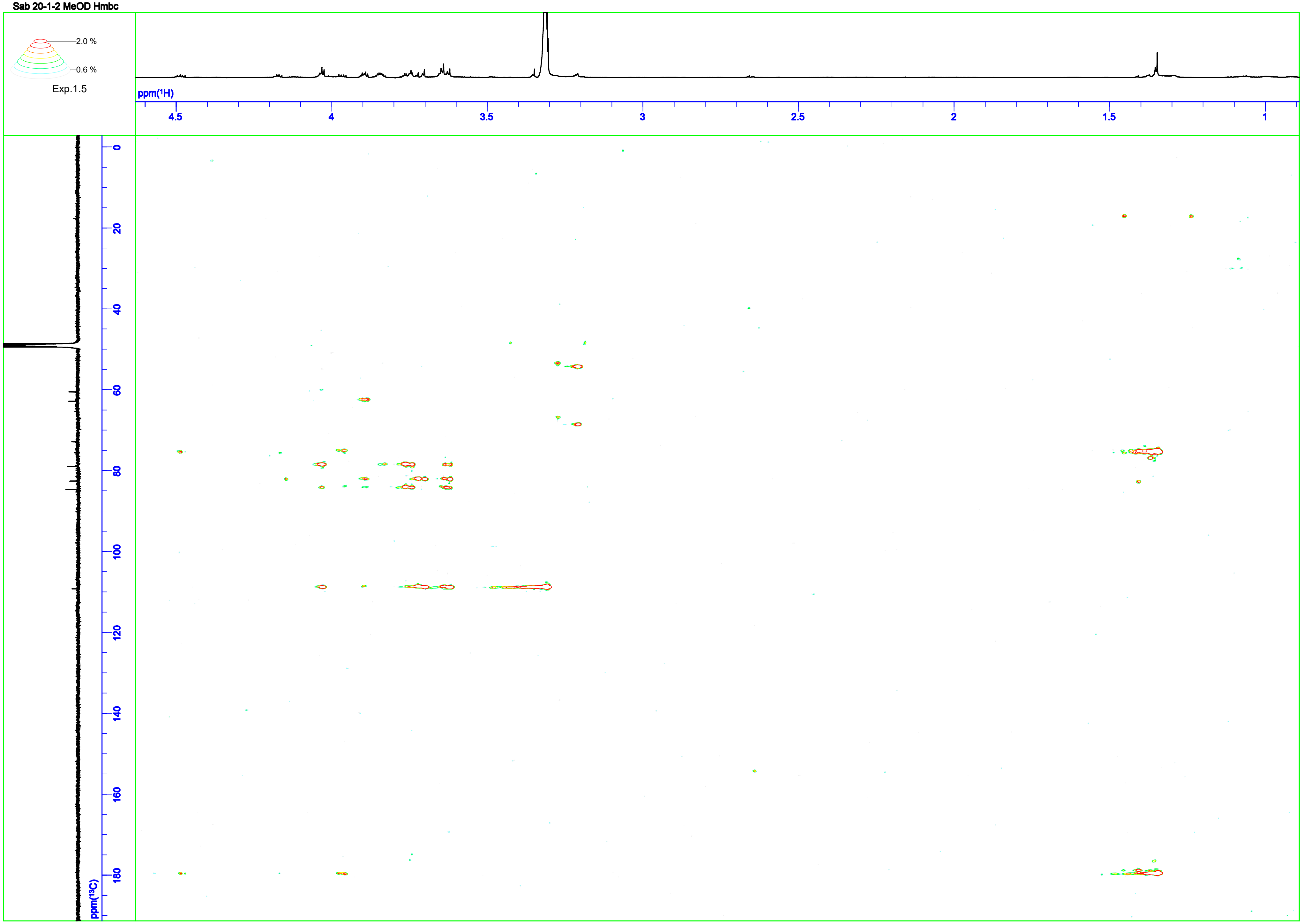

Figure S14 1H NMR of compound 3 in 600 MHz, CD_3OD

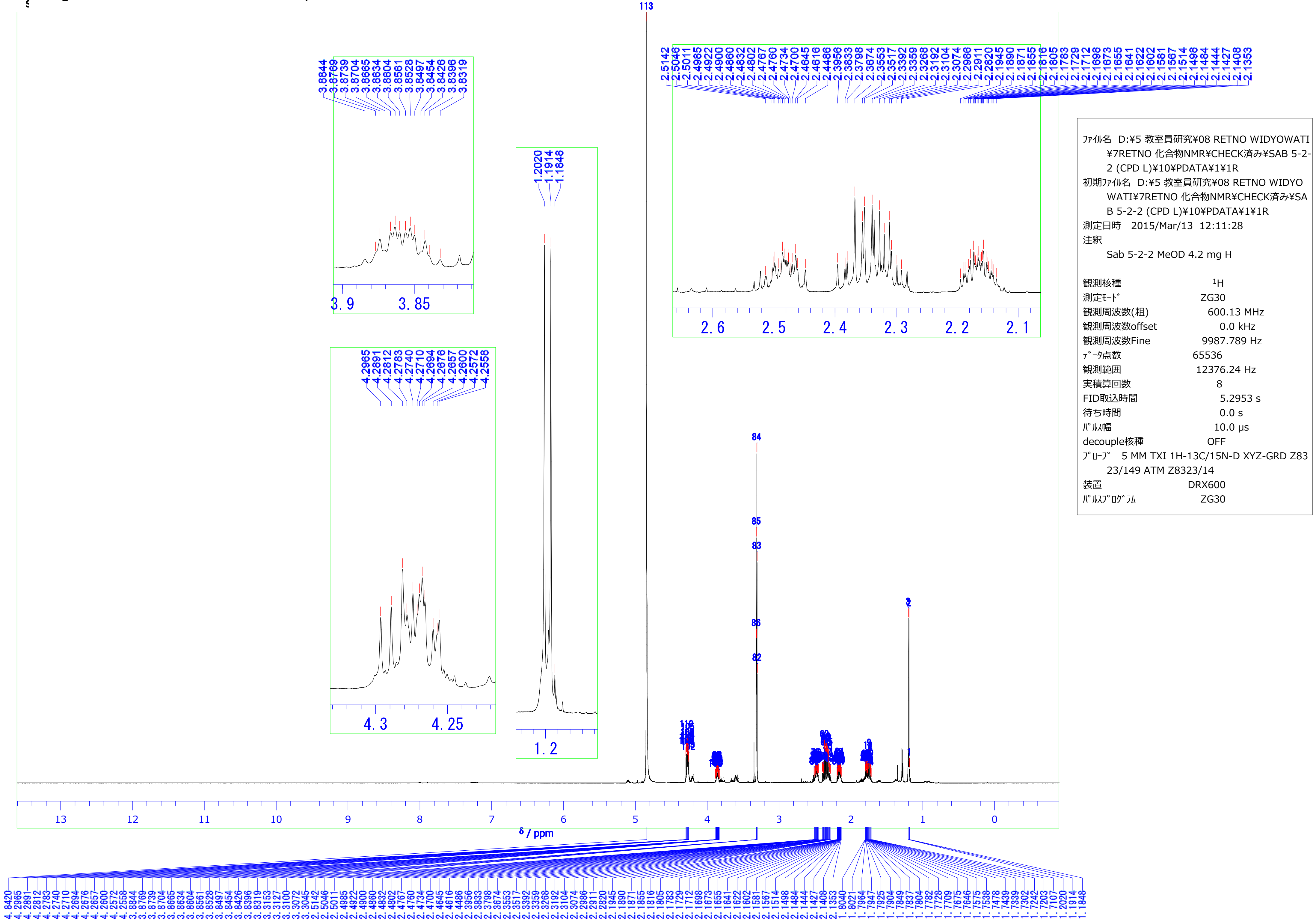

Figure S15 1H NMR (2.31 & 2.37 ppm) of compound 3 in 600 MHz, CD_3OD

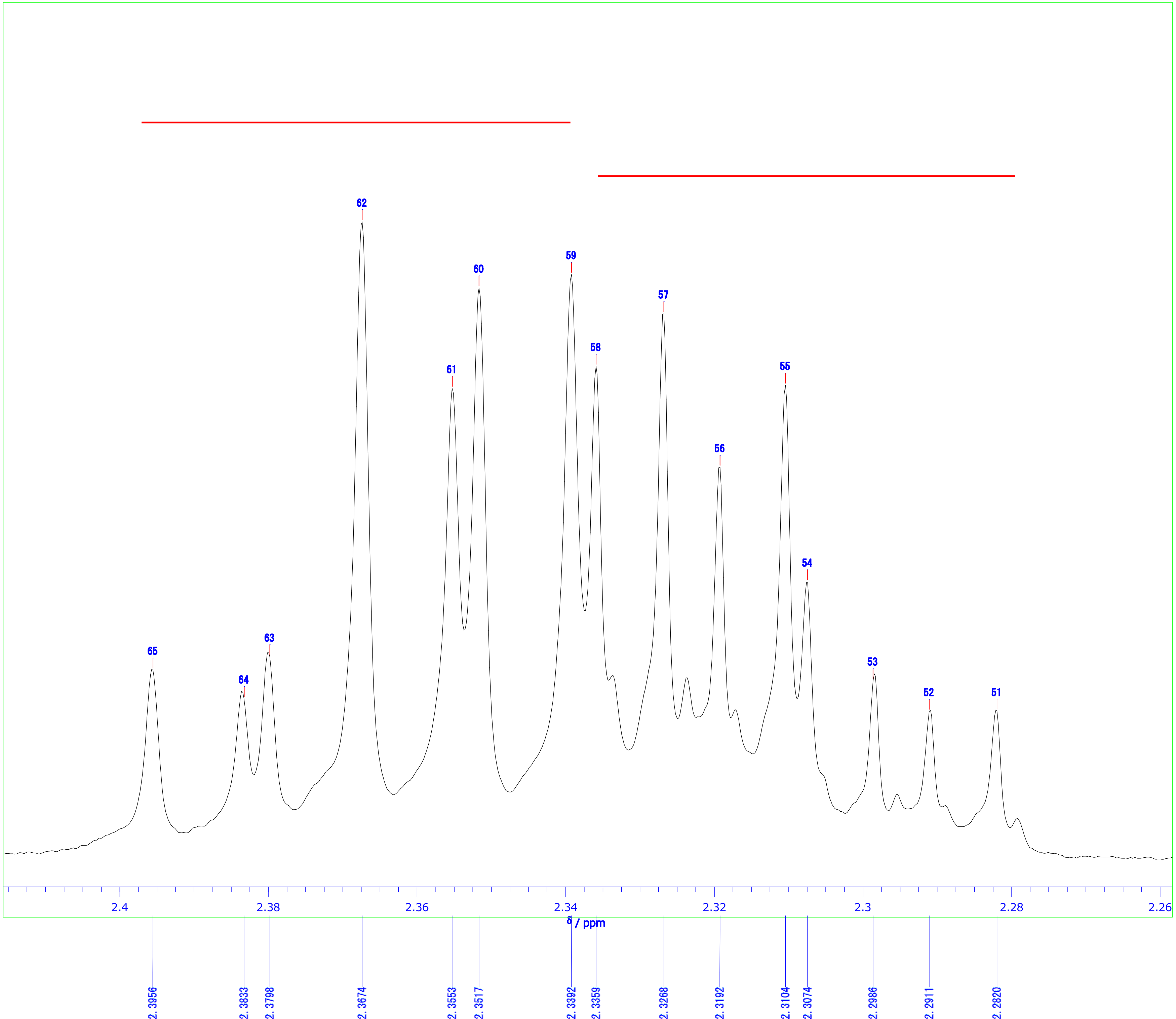

ﾌｧｲﾙ名 D:¥5 教室員研究¥08 RETNO WIDYOWATI ¥7RETNO 化合物NMR¥CHECK済み¥SAB 5-2-2 (CPD L)¥10¥PDATA¥1¥1R
初期ﾌｧｲﾙ名 D:¥5 教室員研究¥08 RETNO WIDYOWATI¥7RETNO 化合物NMR¥CHECK済み¥SAB 5-2-2 (CPD L)¥10¥PDATA¥1¥1R
測定日時 2015/Mar/13 12:11:28
注釈
Sab 5-2-2 MeOD 4.2 mg H

観測核種	^{1}H
測定ﾓｰﾄﾞ	ZG30
観測周波数(粗)	600.13 MHz
観測周波数offset	0.0 kHz
観測周波数Fine	9987.789 Hz
ﾃﾞｰﾀ点数	65536
観測範囲	12376.24 Hz
実積算回数	8
FID取込時間	5.2953 s
待ち時間	0.0 s
ﾊﾟﾙｽ幅	10.0 μs
decouple核種	OFF
ﾌﾟﾛｰﾌﾞ	5 MM TXI 1H-13C/15N-D XYZ-GRD Z8323/149 ATM Z8323/14
装置	DRX600
ﾊﾟﾙｽﾌﾟﾛｸﾞﾗﾑ	ZG30

Figure S16 1H NMR (3.86 ppm) of compound 3 in 600 MHz, CD_3OD

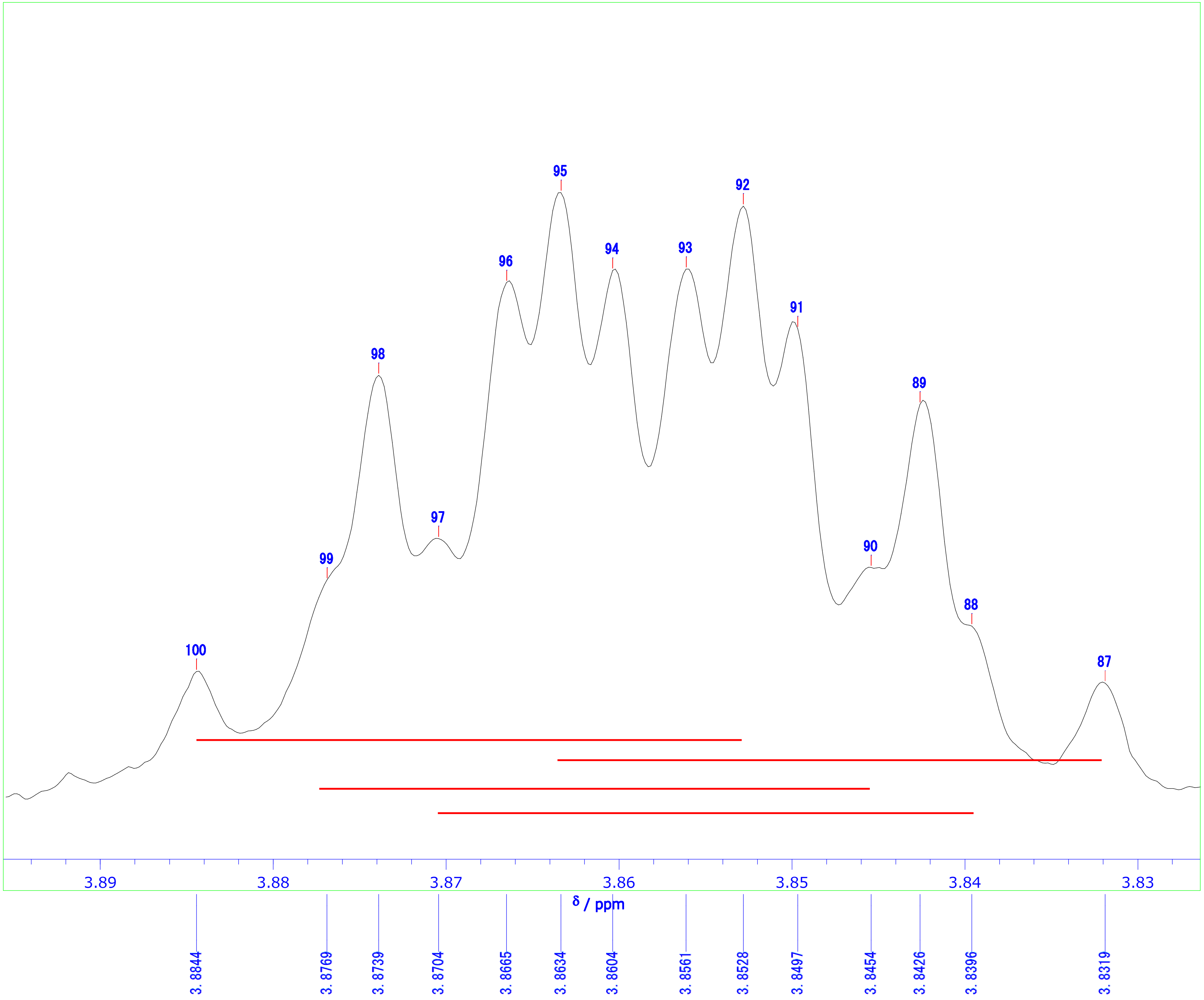

ﾌｧｲﾙ名 D:¥5 教室員研究¥08 RETNO WIDYOWATI¥7RETNO 化合物NMR¥CHECK済み¥SAB 5-2-2 (CPD L)¥1R2.RM1
初期ﾌｧｲﾙ名 D:¥5 教室員研究¥08 RETNO WIDYOWATI¥7RETNO 化合物NMR¥CHECK済み¥SAB 5-2-2 (CPD L)¥10¥PDATA¥1¥1R
測定日時 13/Mar/2015 12:11:28
注釈
Sab 5-2-2 MeOD 4.2 mg H

観測核種	^{1}H
測定ﾓｰﾄﾞ	ZG30
観測周波数(粗)	600.13 MHz
観測周波数offset	0.0 kHz
観測周波数Fine	9987.789 Hz
ﾃﾞｰﾀ点数	65536
観測範囲	12376.24 Hz
実積算回数	8
FID取込時間	5.2953 s
待ち時間	0.0 s
ﾊﾟﾙｽ幅	10.0 μs
decouple核種	NUL
ﾌﾟﾛｰﾌﾞ	5 MM TXI 1H-13C/15N-D XYZ-GRD Z8
装置	DRX600
ﾊﾟﾙｽﾌﾟﾛｸﾞﾗﾑ	ZG30
Gradientﾌﾟﾛｸﾞﾗﾑ	
試料温度	26.85 ℃
測定溶媒	MEOD
Chemical shift参照値	3.31 ppm

Figure S17 1H NMR (4.27 & 4.29 ppm) of compound 3 in 600 MHz, CD_3OD

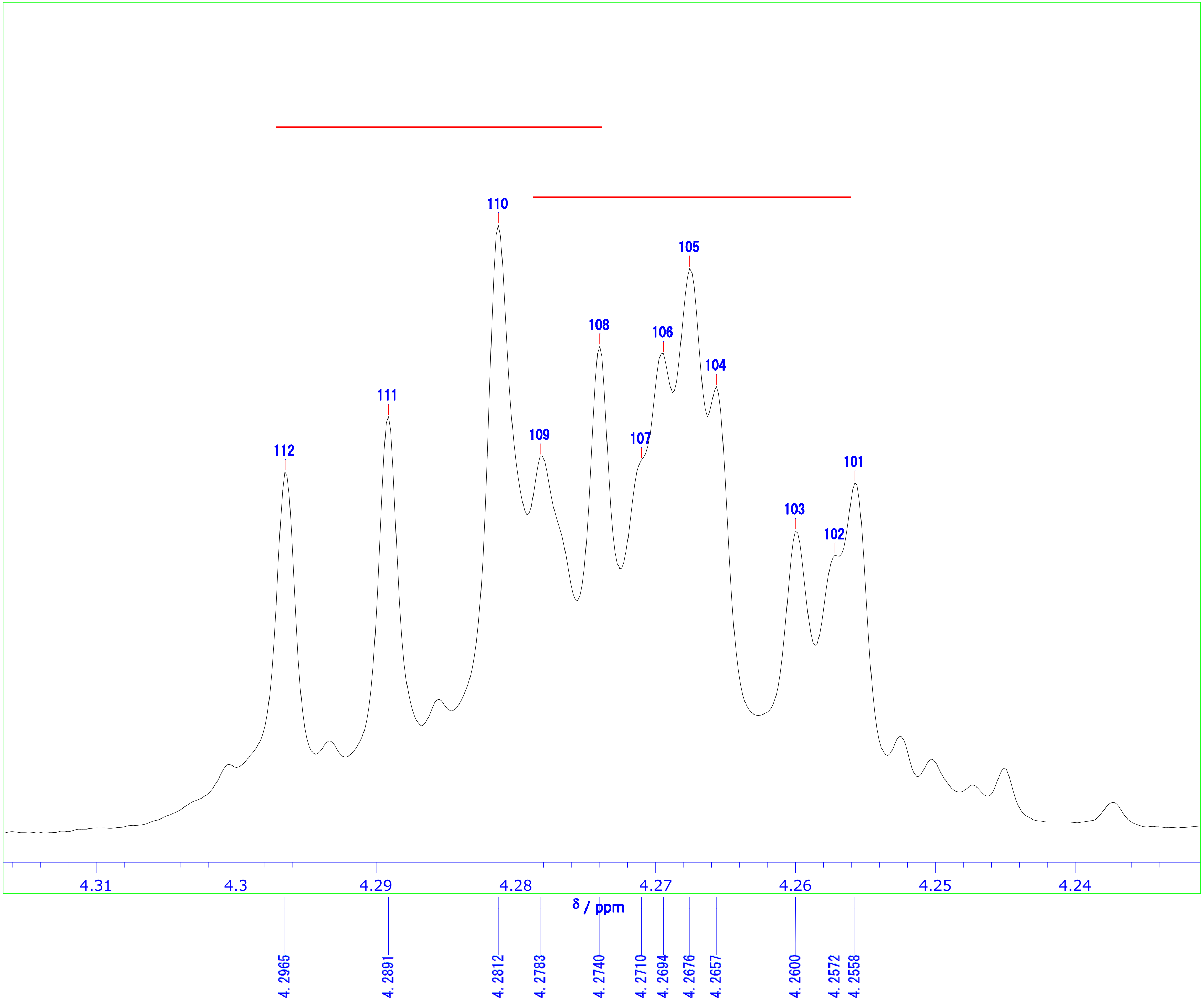

ﾌｧｲﾙ名	D:¥5 教室員研究¥08 RETNO WIDYOWATI¥7RETNO 化合物NMR¥CHECK済み¥SAB 5-2-2 (CPD L)¥1R2.RM1
初期ﾌｧｲﾙ名	D:¥5 教室員研究¥08 RETNO WIDYOWATI¥7RETNO 化合物NMR¥CHECK済み¥SAB 5-2-2 (CPD L)¥10¥PDATA¥1¥1R
測定日時	13/Mar/2015 12:11:28
注釈	Sab 5-2-2 MeOD 4.2 mg H
観測核種	^{1}H
測定ﾓｰﾄﾞ	ZG30
観測周波数(粗)	600.13 MHz
観測周波数offset	0.0 kHz
観測周波数Fine	9987.789 Hz
ﾃﾞｰﾀ点数	65536
観測範囲	12376.24 Hz
実積算回数	8
FID取込時間	5.2953 s
待ち時間	0.0 s
ﾊﾟﾙｽ幅	10.0 μs
decouple核種	NUL
ﾌﾟﾛｰﾌﾞ	5 MM TXI 1H-13C/15N-D XYZ-GRD Z8
装置	DRX600
ﾊﾟﾙｽﾌﾟﾛｸﾞﾗﾑ	ZG30
Gradientﾌﾟﾛﾌﾗﾑ	
試料温度	26.85 ℃
測定溶媒	MEOD
Chemical shift参照値	3.31 ppm

Figure S18 13C NMR of compound 3 in 150 MHz, CD_3OD

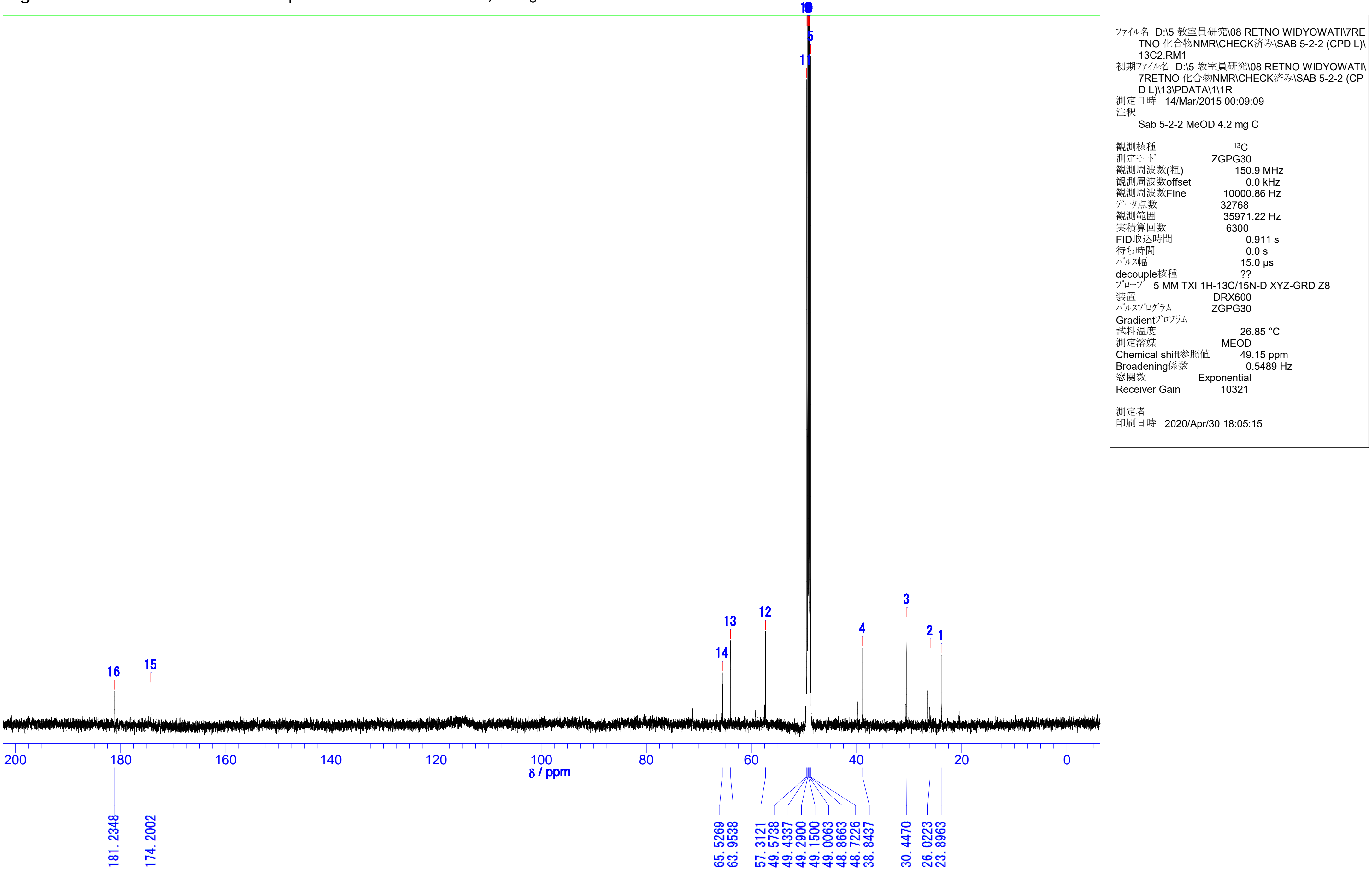

Figure S19 COSY of compound 3 in 600 MHz, CD_3OD

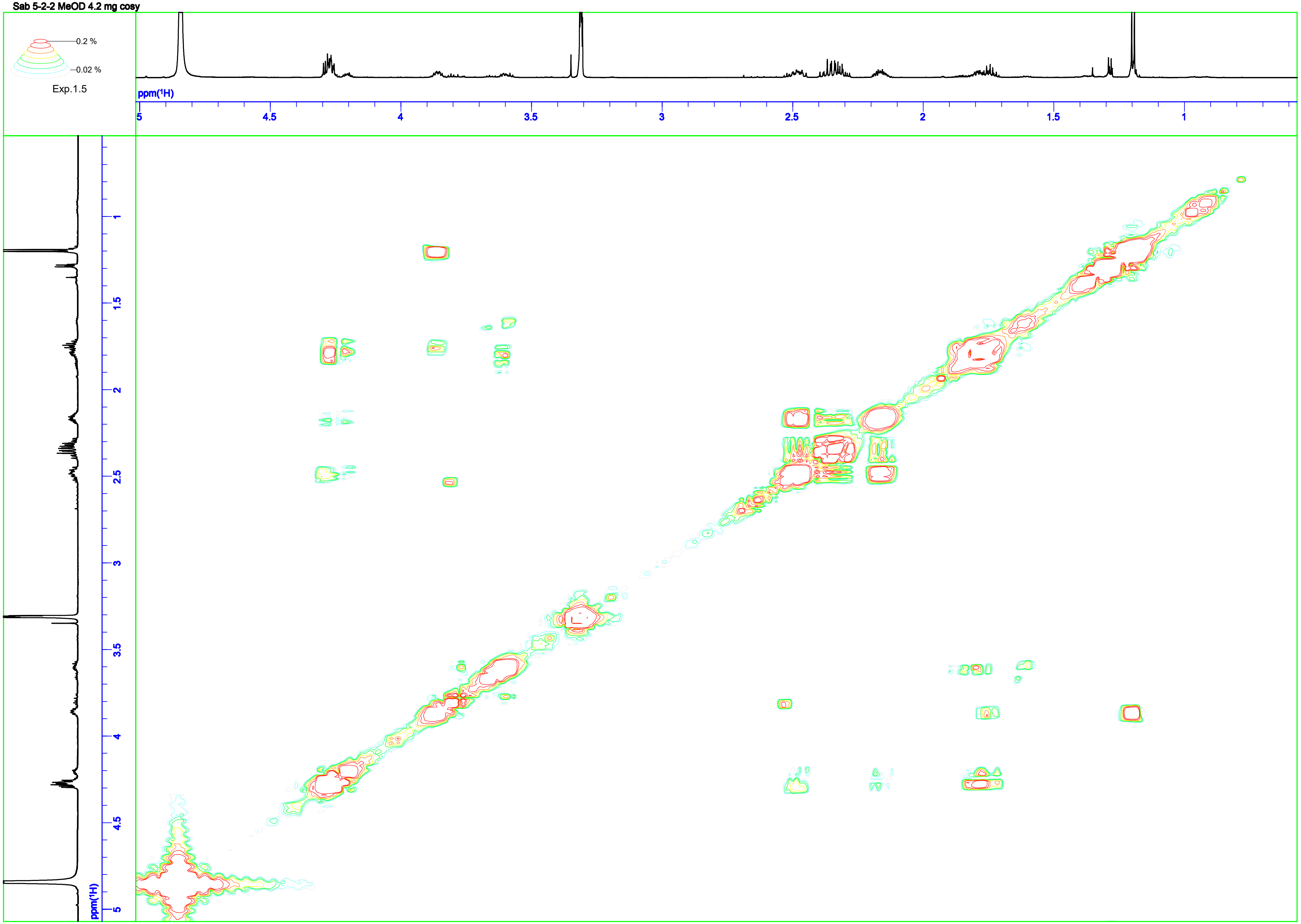

Figure S20 HSQC of compound 3 in 150 and 600 MHz, CD_3OD

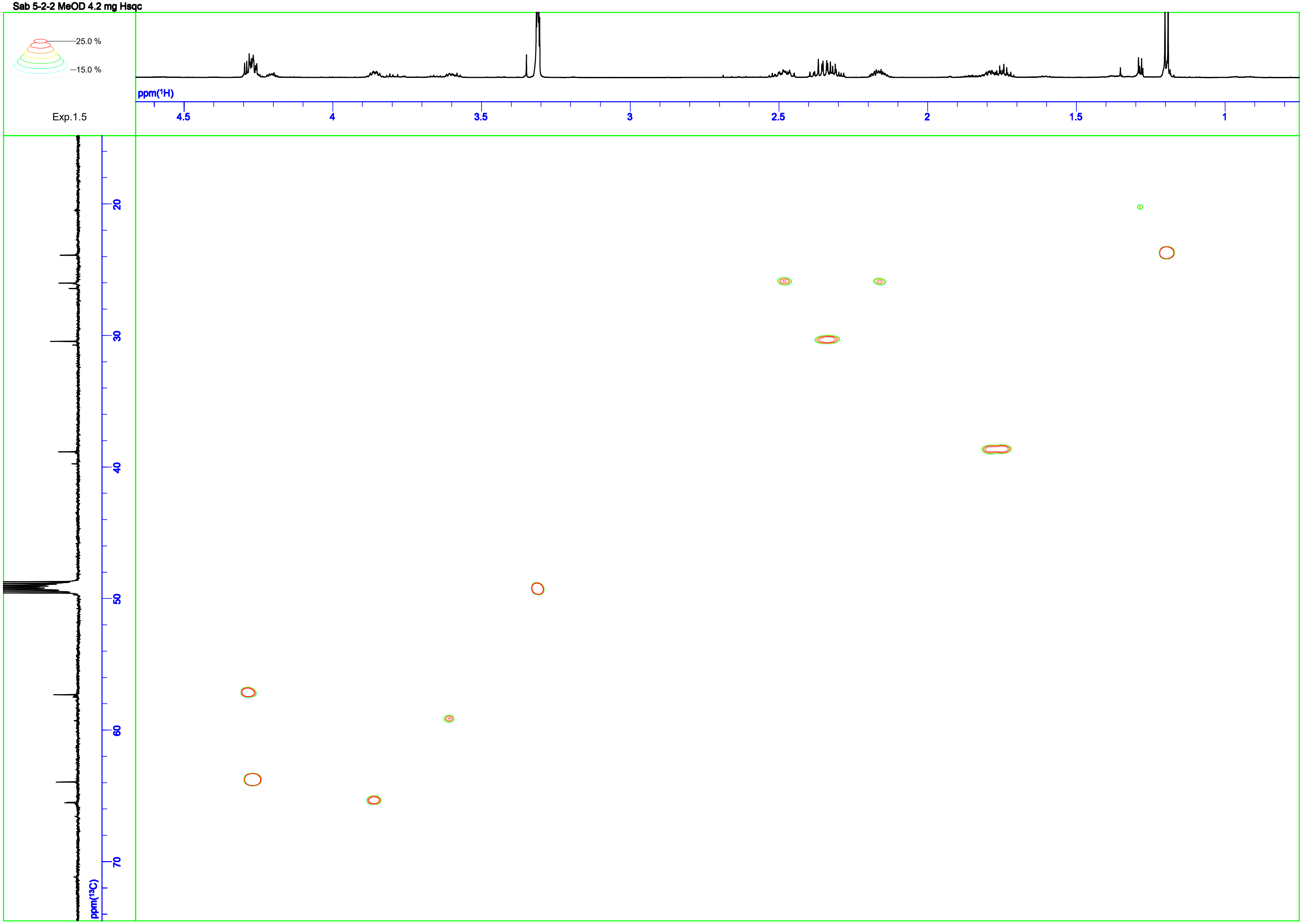

Figure S21 HMBC of compound 3 in 150 and 600 MHz, CD_3OD

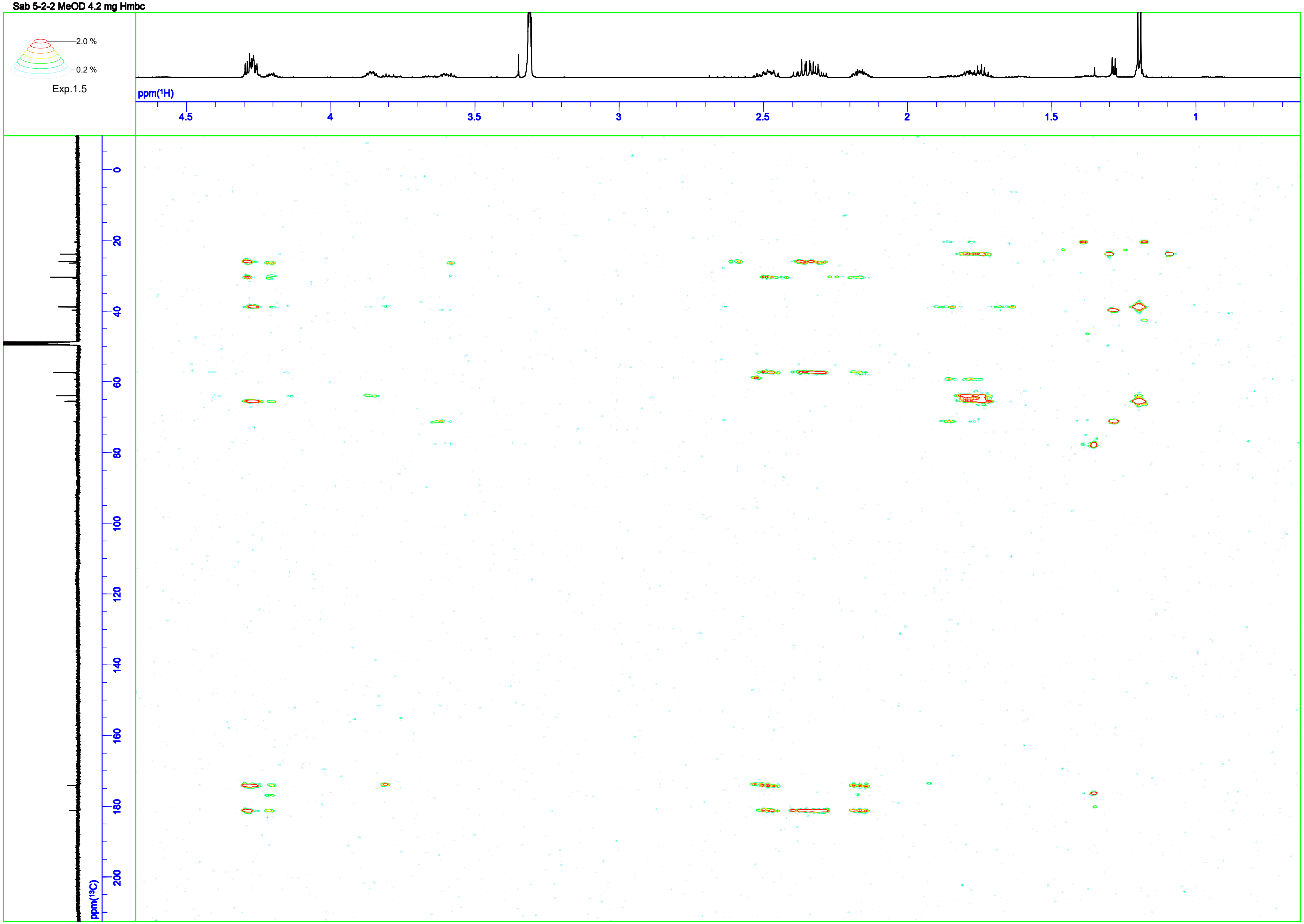

Figure S22 1H NMR of compound 4 in 600 MHz, CD_3OD

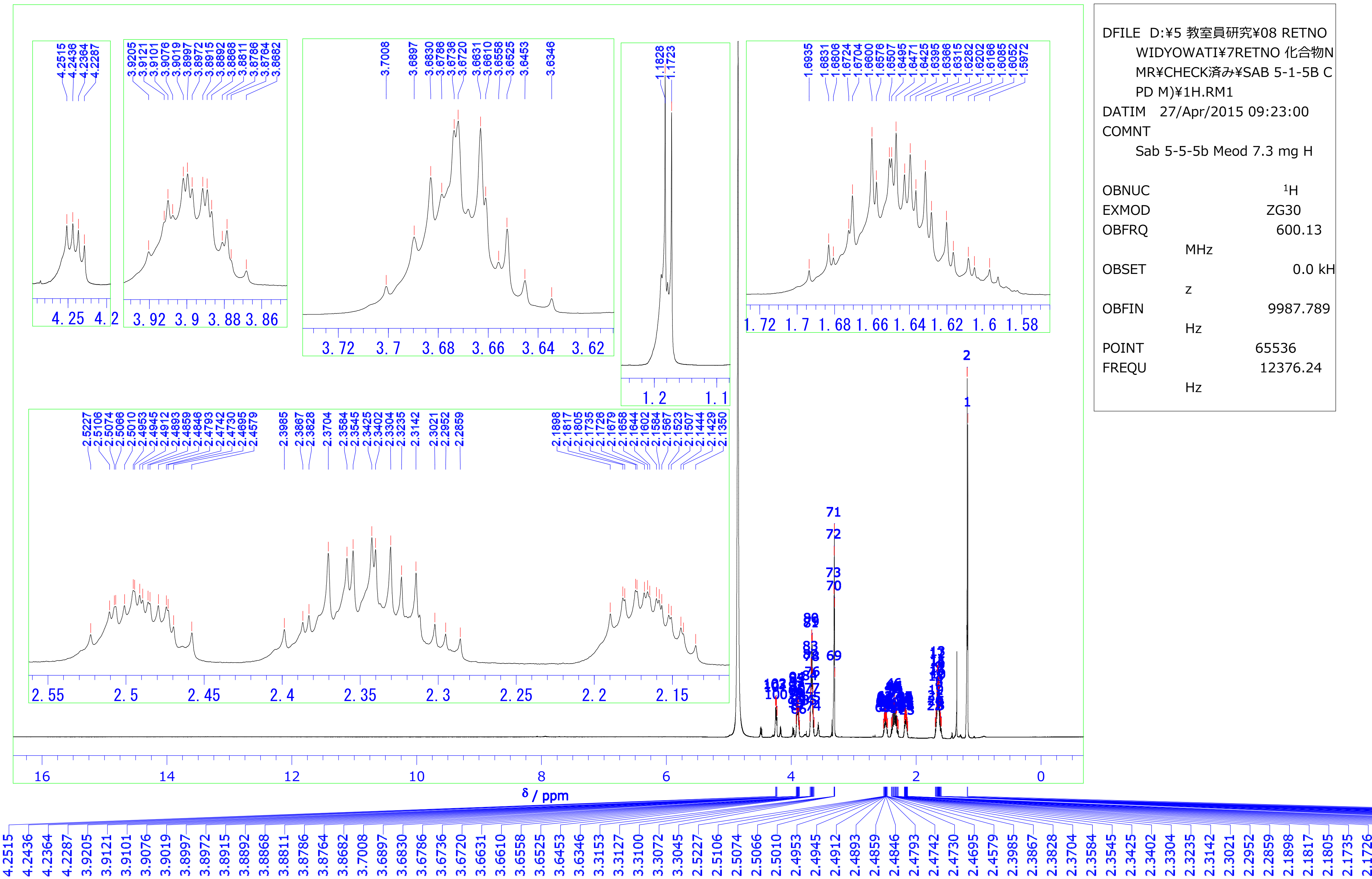

Figure S23 1H NMR (1.62 & 1.67 ppm) of compound 4 in 600 MHz, CD_3OD

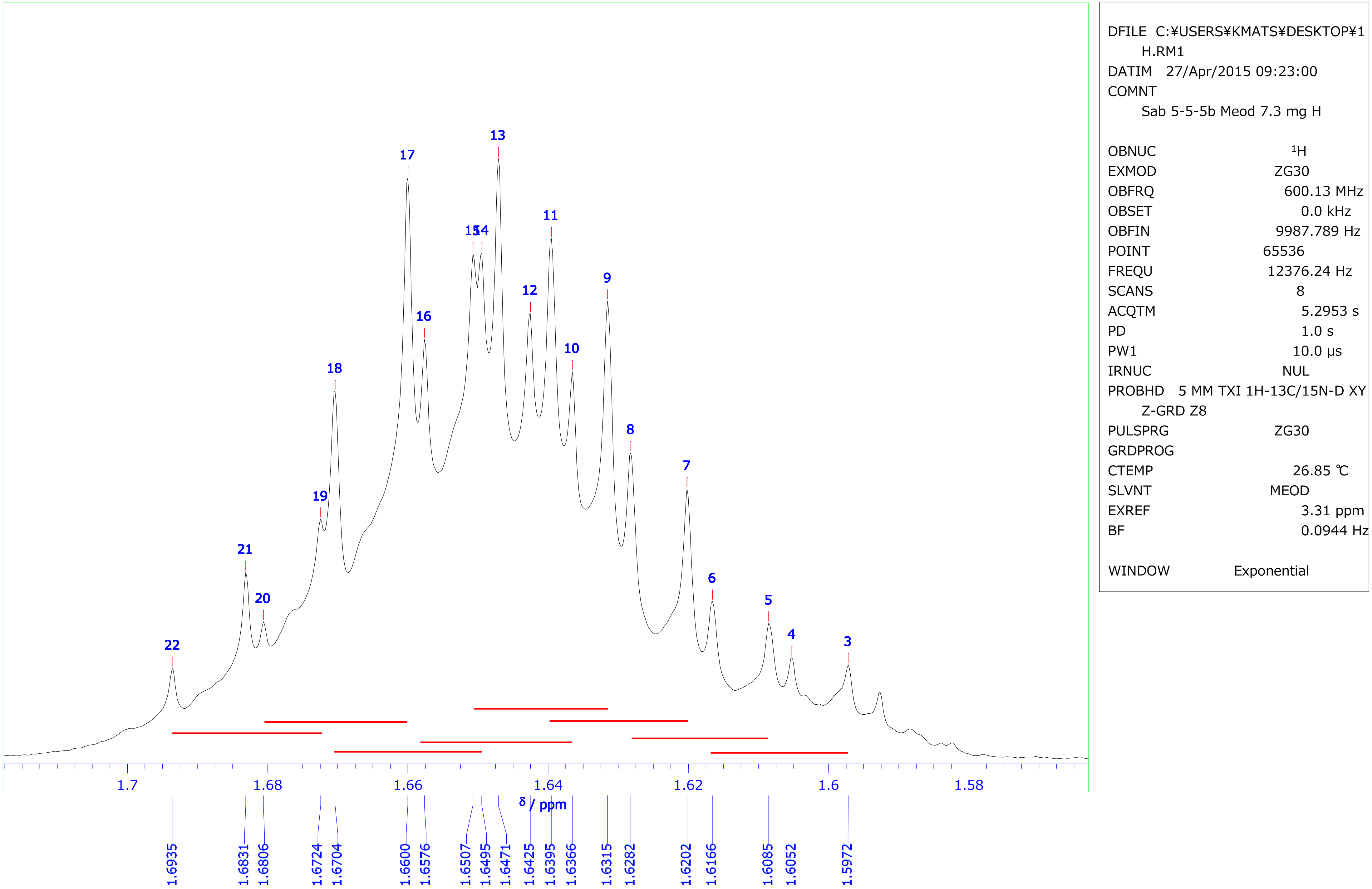

Figure S24 1H NMR (2.17 ppm) of compound 4 in 600 MHz, CD_3OD

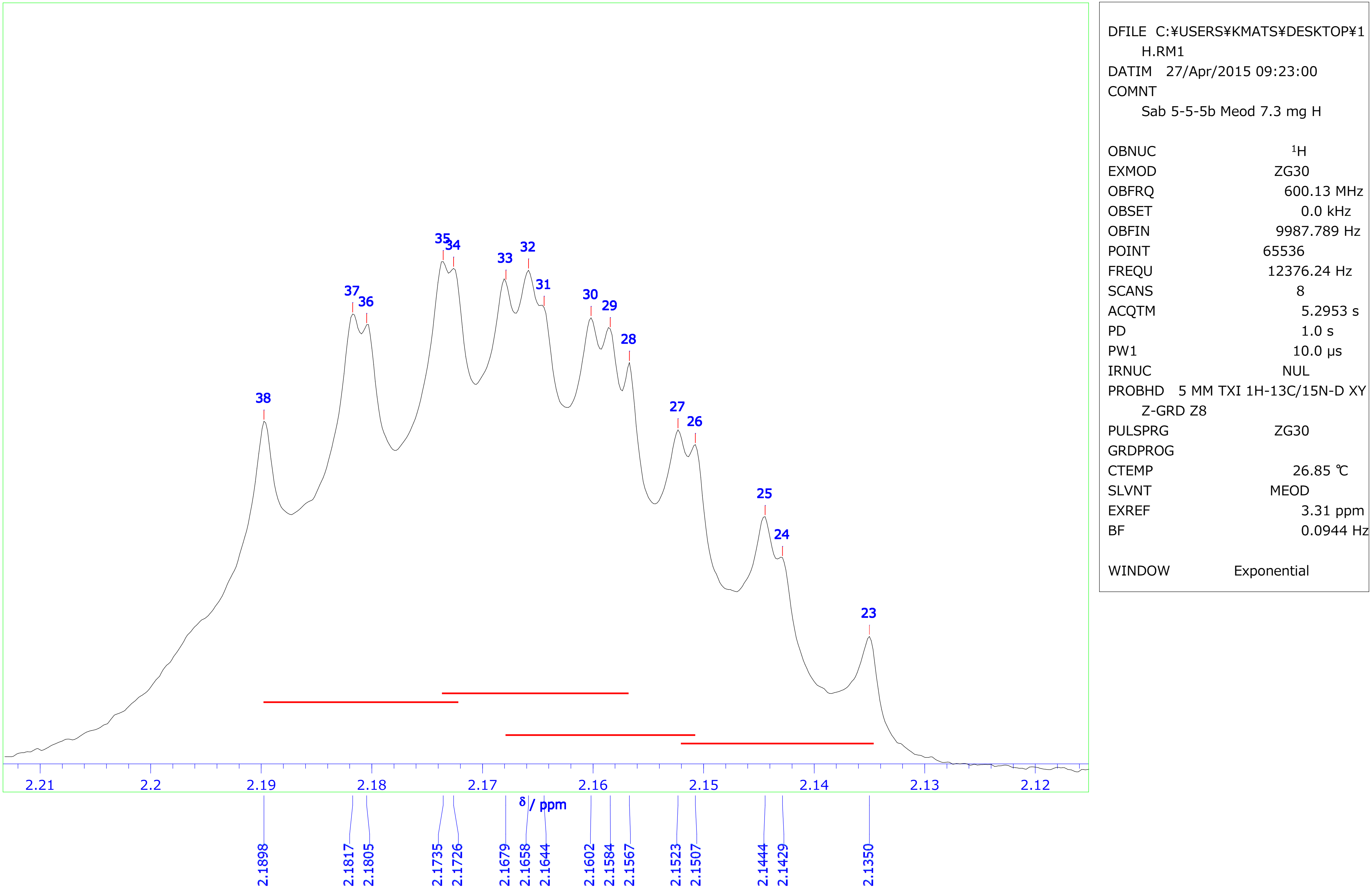

Figure S25 1H NMR (2.31 & 2.37 ppm) of compound 4 in 600 MHz, CD_3OD

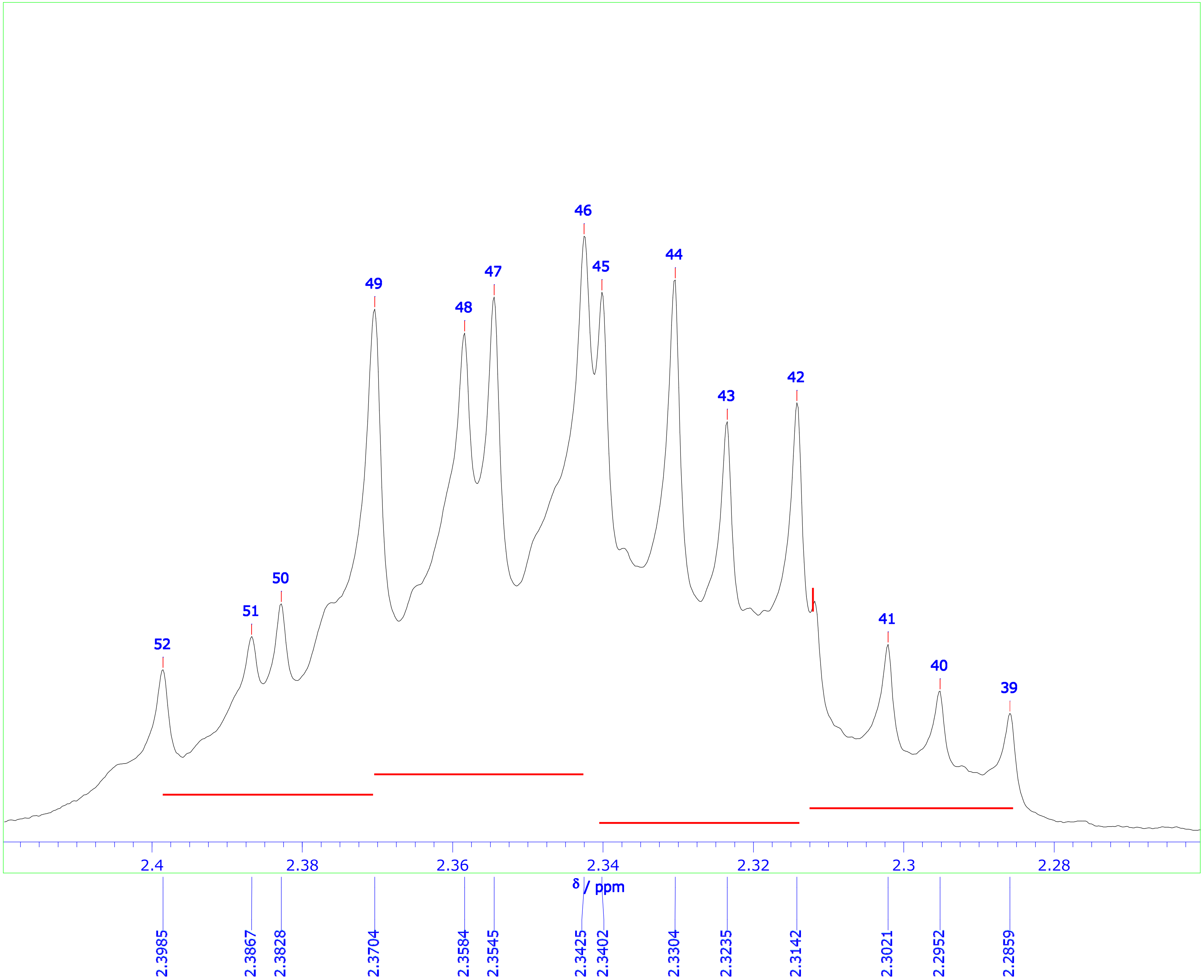

DFILE C:¥USERS¥KMATS¥DESKTOP¥1 H.RM1
DATIM 27/Apr/2015 09:23:00
COMNT
Sab 5-5-5b Meod 7.3 mg H

OBNUC	^1H
EXMOD	ZG30
OBFRQ	600.13 MHz
OBSET	0.0 kHz
OBFIN	9987.789 Hz
POINT	65536
FREQU	12376.24 Hz
SCANS	8
ACQTM	5.2953 s
PD	1.0 s
PW1	10.0 μs
IRNUC	NUL
PROBHD	5 MM TXI 1H-13C/15N-D XY Z-GRD Z8
PULSPRG	ZG30
GRDPROG	
CTEMP	26.85 ℃
SLVNT	MEOD
EXREF	3.31 ppm
BF	0.0944 Hz
WINDOW	Exponential

Figure S26 1H NMR (2.49 ppm) of compound 4 in 600 MHz, CD_3OD

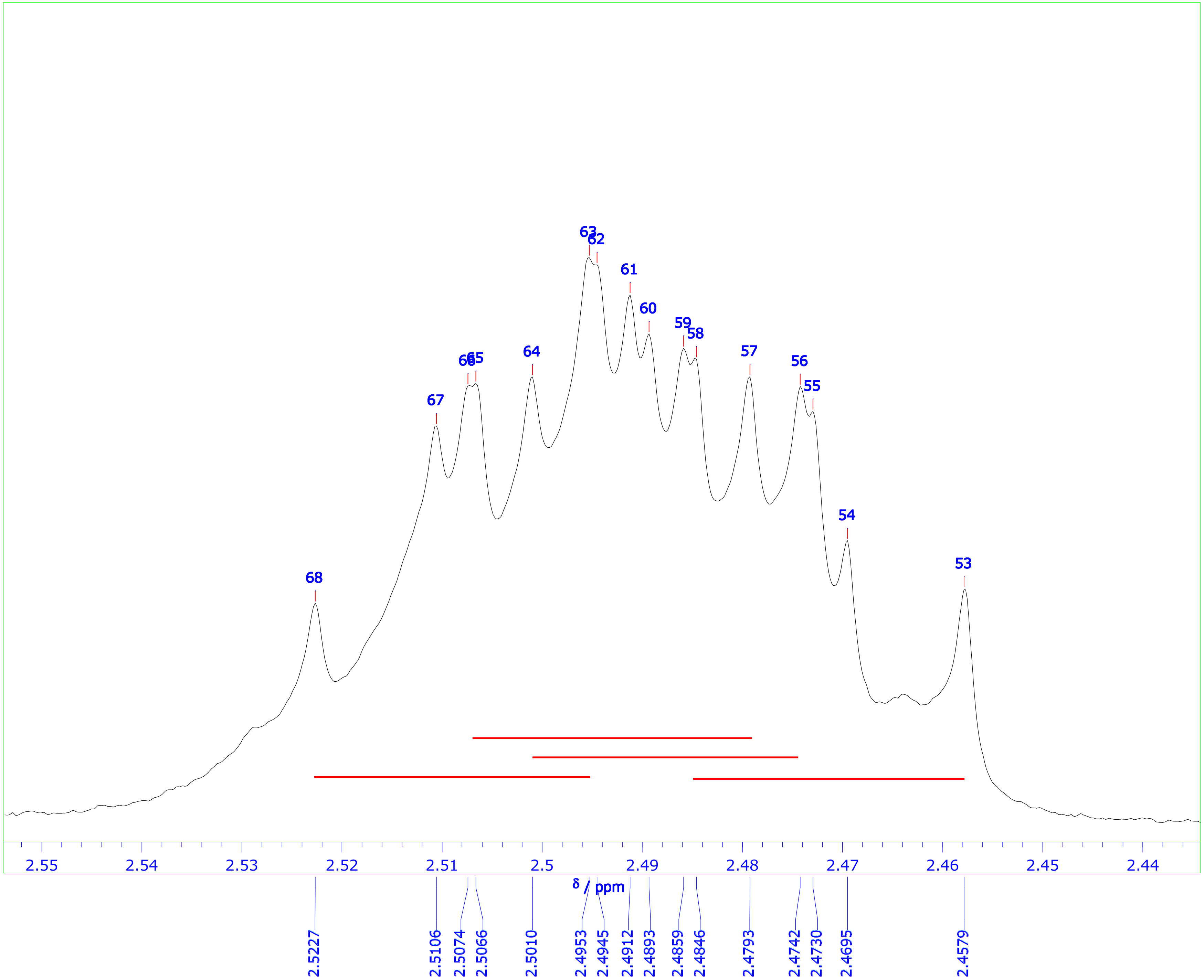

DFILE C:¥USERS¥KMATS¥DESKTOP¥1H.RM1
DATIM 27/Apr/2015 09:23:00
COMNT
Sab 5-5-5b Meod 7.3 mg H

OBNUC	^{1}H
EXMOD	ZG30
OBFRQ	600.13 MHz
OBSET	0.0 kHz
OBFIN	9987.789 Hz
POINT	65536
FREQU	12376.24 Hz
SCANS	8
ACQTM	5.2953 s
PD	1.0 s
PW1	10.0 μs
IRNUC	NUL
PROBHD	5 MM TXI 1H-13C/15N-D XYZ-GRD Z8
PULSPRG	ZG30
GRDPROG	
CTEMP	26.85 ℃
SLVNT	MEOD
EXREF	3.31 ppm
BF	0.0944 Hz
WINDOW	Exponential

Figure S27 1H NMR (3.65 & 3.68 ppm) of compound 4 in 600 MHz, CD_3OD

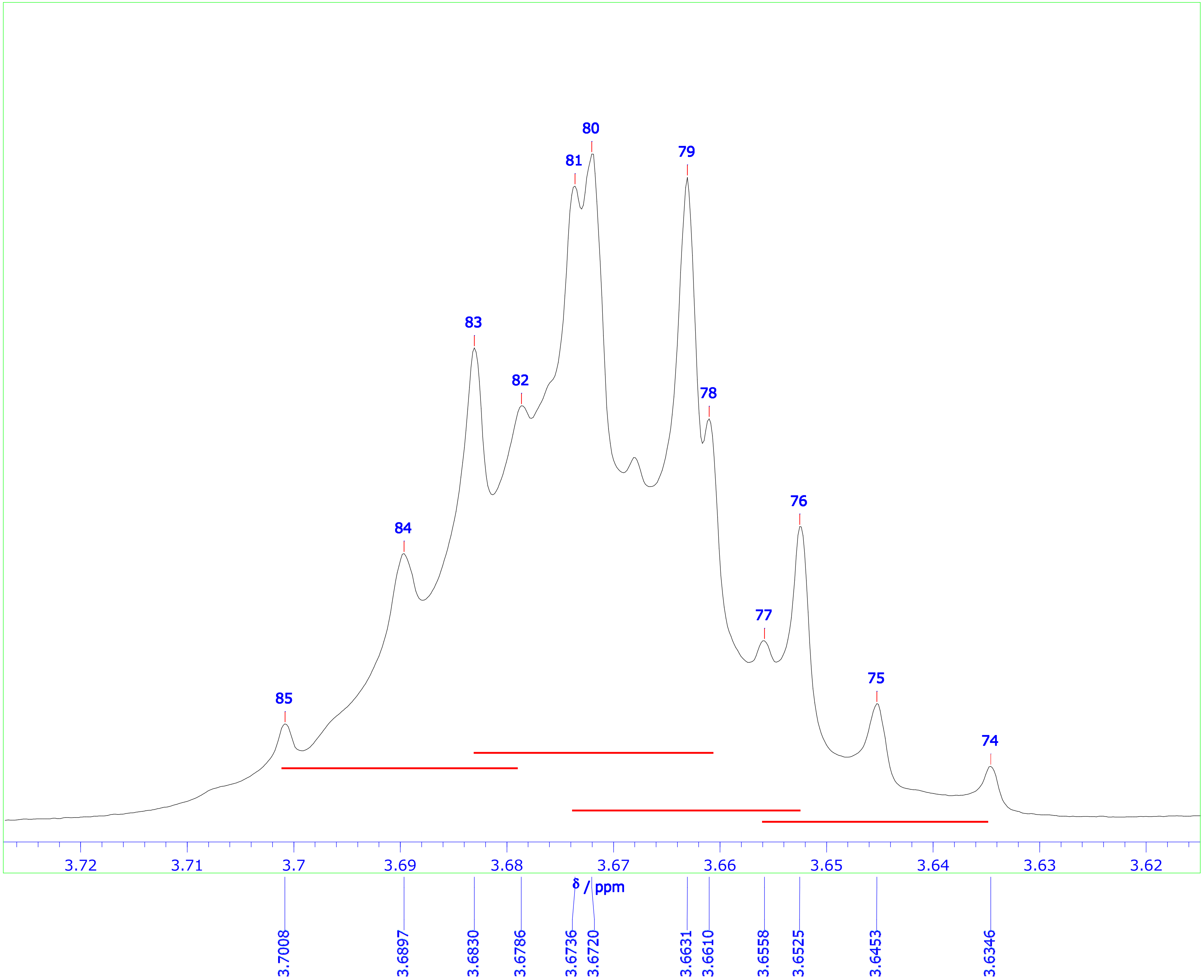

DFILE C:¥USERS¥KMATS¥DESKTOP¥1H.RM1
DATIM 27/Apr/2015 09:23:00
COMNT Sab 5-5-5b Meod 7.3 mg H

OBNUC	^1H
EXMOD	ZG30
OBFRQ	600.13 MHz
OBSET	0.0 kHz
OBFIN	9987.789 Hz
POINT	65536
FREQU	12376.24 Hz
SCANS	8
ACQTM	5.2953 s
PD	1.0 s
PW1	10.0 μs
IRNUC	NUL
PROBHD	5 MM TXI 1H-13C/15N-D XYZ-GRD Z8
PULSPRG	ZG30
GRDPROG	
CTEMP	26.85 ℃
SLVNT	MEOD
EXREF	3.31 ppm
BF	0.0944 Hz
WINDOW	Exponential

Figure S28 1H NMR (3.89 ppm) of compound 4 in 600 MHz, CD_3OD

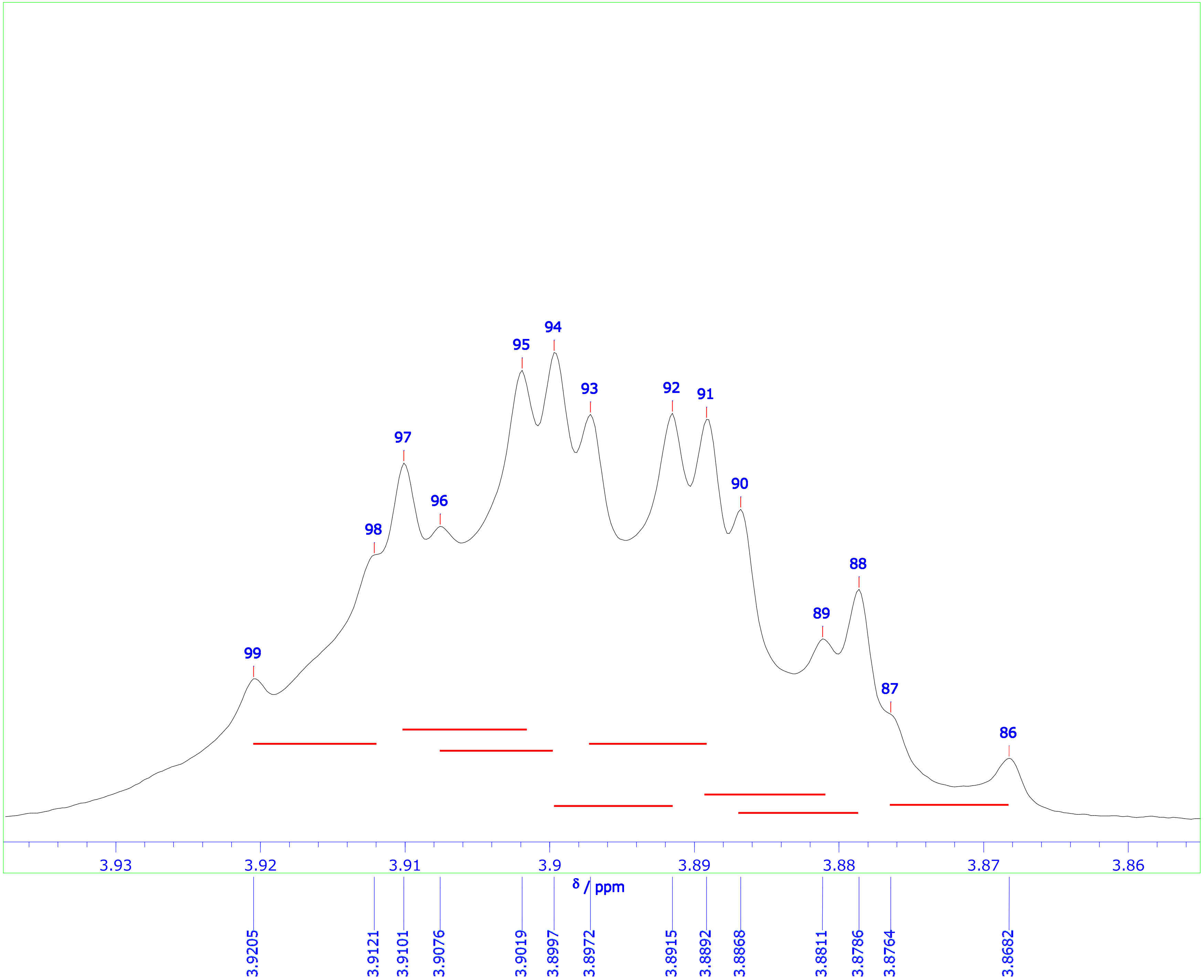

DFILE D:¥5 教室員研究¥08 RETNO WIDYOWATI¥7RETNO 化合物NMR¥CHECK済み¥SAB 5-1-5B CPD M)¥1H.RM1
DATIM 27/Apr/2015 09:23:00
COMNT
Sab 5-5-5b Meod 7.3 mg H

OBNUC	^{1}H
EXMOD	ZG30
OBFRQ	600.13 MHz
OBSET	0.0 kHz
OBFIN	9987.789 Hz
POINT	65536
FREQU	12376.24 Hz
SCANS	8
ACQTM	5.2953 s
PD	1.0 s
PW1	10.0 μs
IRNUC	??
PROBHD	5 MM TXI 1H-13C/15N-D XYZ-GRD Z8
PULSPRG	ZG30
GRDPROG	
CTEMP	26.85 ℃
SLVNT	MEOD
EXREF	3.31 ppm
BF	0.0944 Hz

Figure S29 13C NMR of compound 4 in 150 MHz, CD_3OD

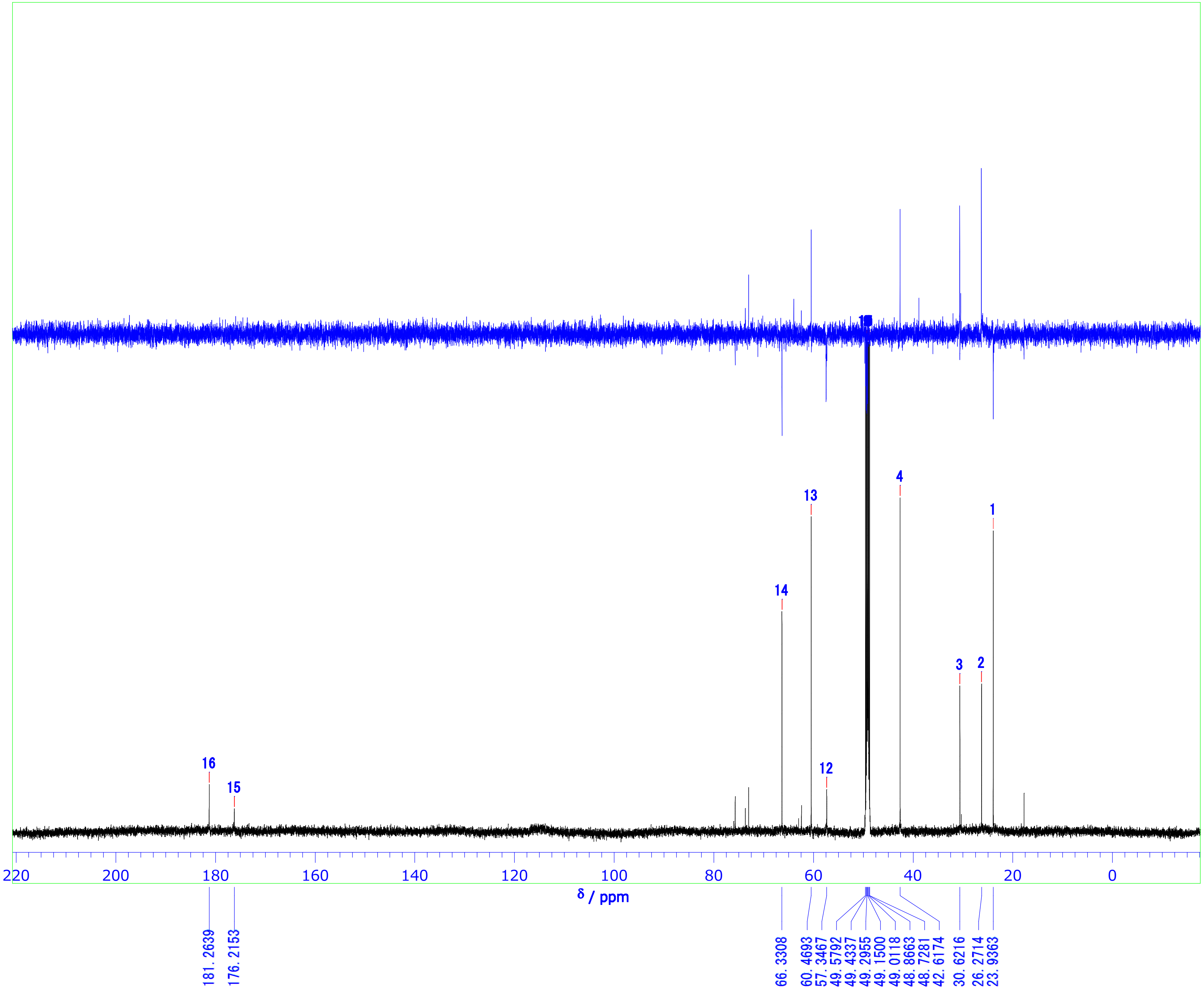

ﾌｧｲﾙ名 D:¥5 教室員研究¥08 RETNO WIDYOWATI¥7RETNO 化合物NMR¥CHECK済み¥SAB 5-1-5B CPD M)¥13C&DEPT.RM1
初期ﾌｧｲﾙ名 D:¥5 教室員研究¥08 RETNO WIDYOWATI¥7RETNO 化合物NMR¥CHECK済み¥SAB 5-1-5B CPD M)¥23 13C¥PDATA¥1¥1R
測定日時 17/Apr/2015 00:05:05
注釈
Sab 5-1-5b MeOD 7.3 mg c

観測核種	^{13}C
測定ﾓｰﾄﾞ	ZGPG30
観測周波数(粗)	150.9 MHz
観測周波数offset	0.0 kHz
観測周波数Fine	10000.86 Hz
ﾃﾞｰﾀ点数	32768
観測範囲	35971.22 Hz
実積算回数	5759
FID取込時間	0.911 s
待ち時間	0.0 s
ﾊﾟﾙｽ幅	15.0 μs
decouple核種	??
ﾌﾟﾛｰﾌﾞ	5 MM TXI 1H-13C/15N-D XYZ-GRD Z8
装置	DRX600
ﾊﾟﾙｽﾌﾟﾛｸﾞﾗﾑ	ZGPG30
Gradientﾌﾟﾛｸﾞﾗﾑ	
試料温度	26.85 ℃
測定溶媒	MEOD
Chemical shift参照値	49.15 ppm
Broadening係数	0.5489 H

Figure S30 COSY of compound 4 in 600 MHz, CD_3OD

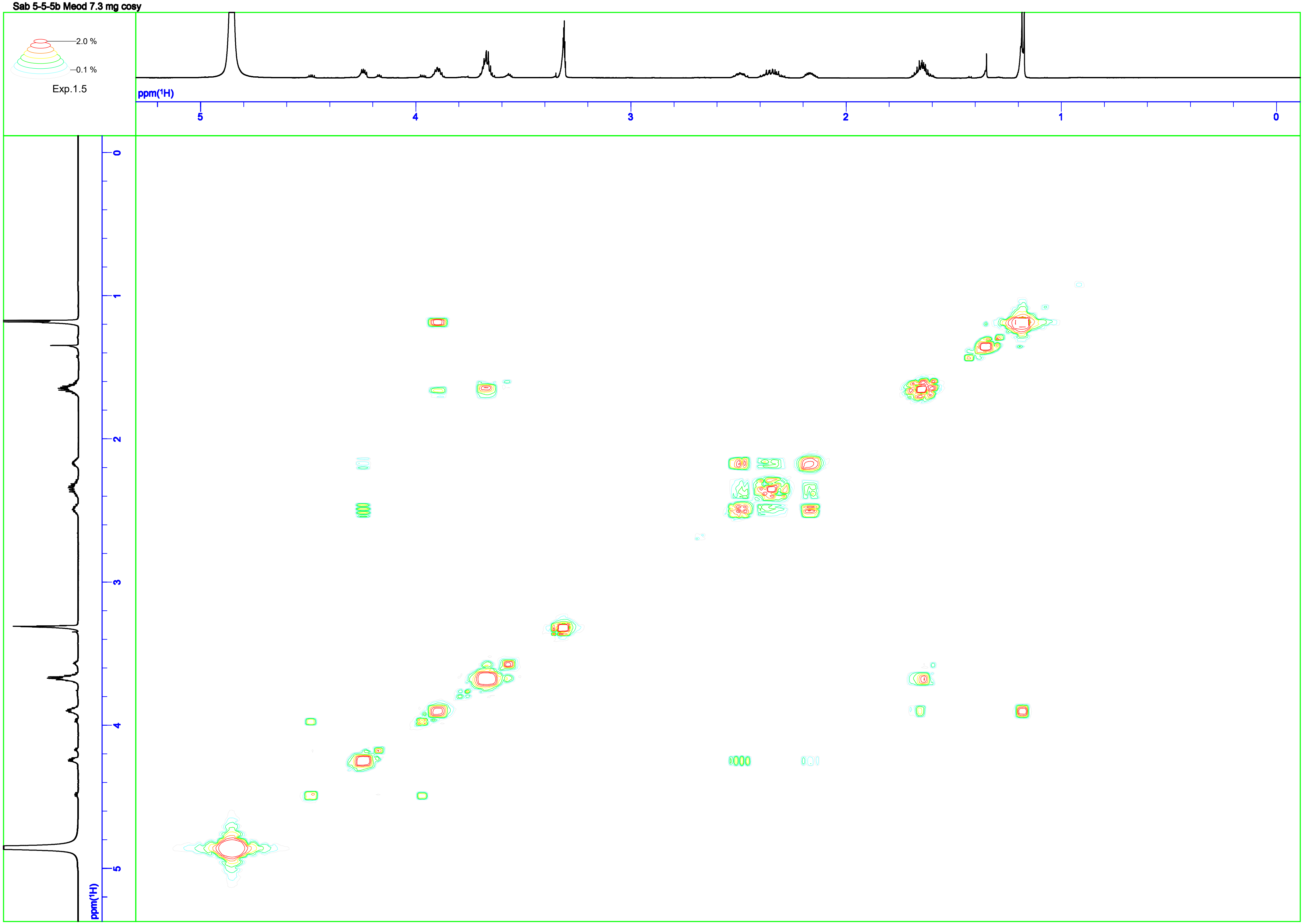

Figure S31 HSQC of compound 4 in 150 and 600 MHz, CD_3OD

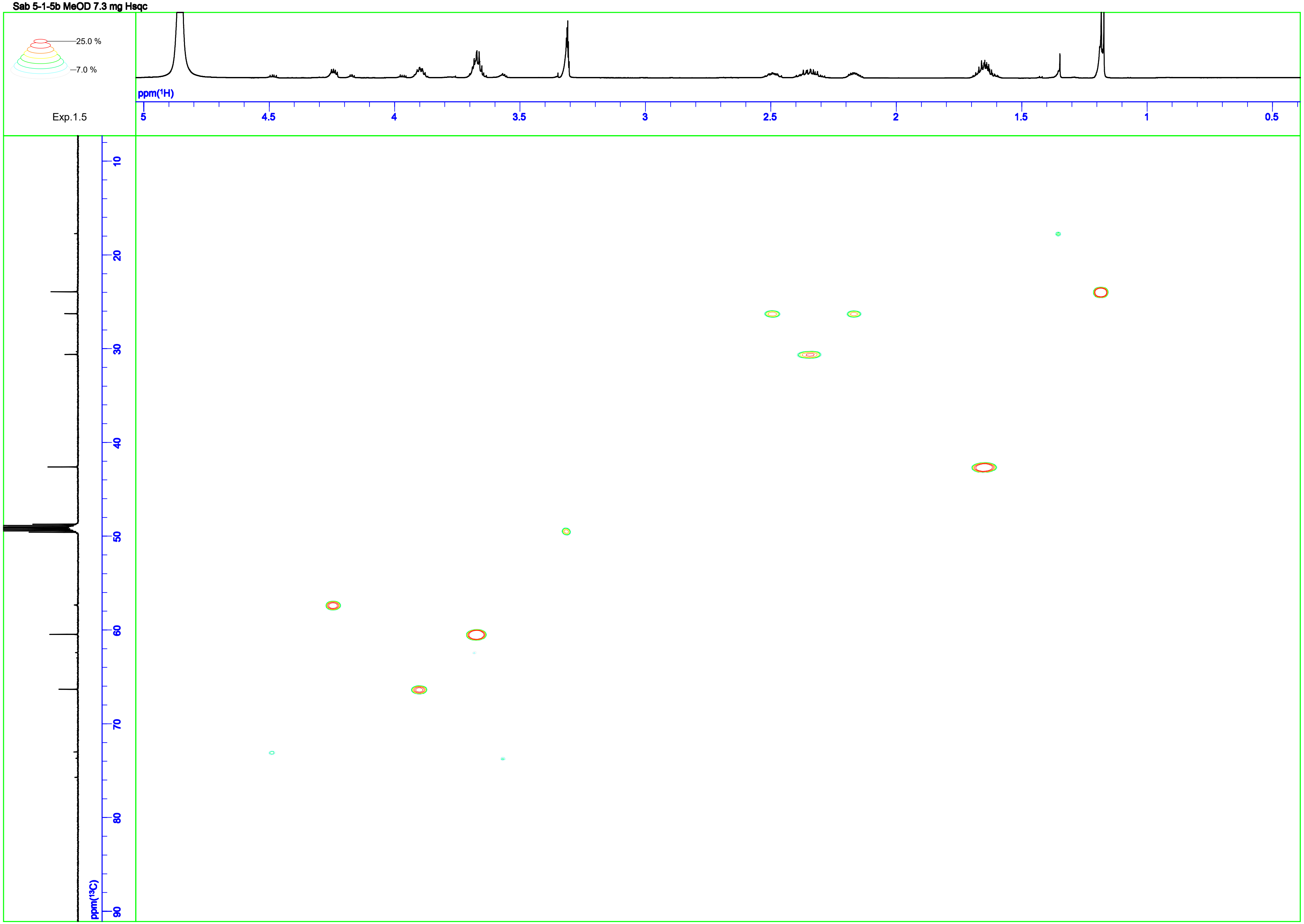

Figure S32 HMBC of compound 4 in 150 and 600 MHz, CD_3OD

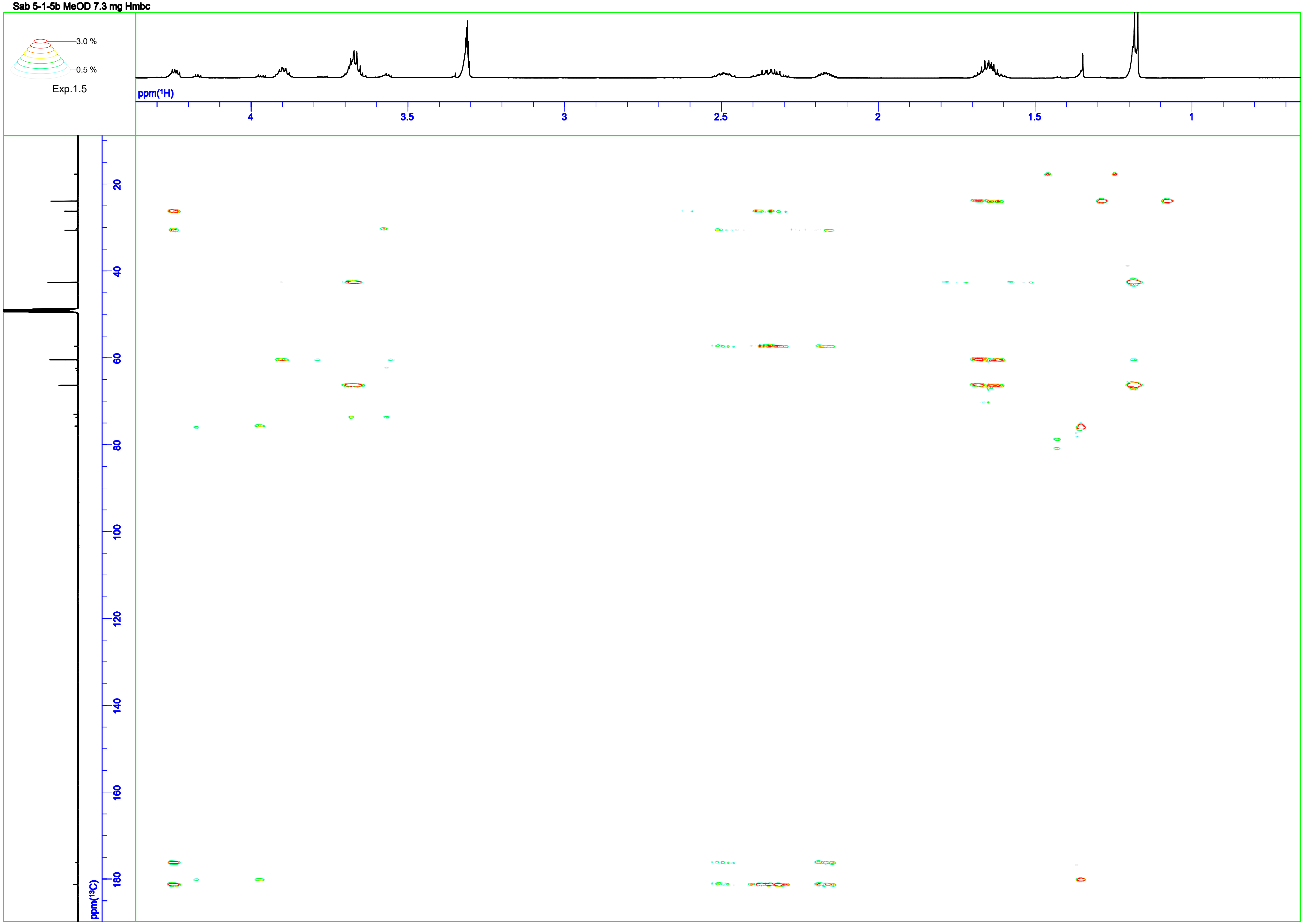

Supplement: Supplementary file 1 [file molecules-25-02500-s001.pdf]
